# Deciphering immune heterogeneity in lung adenocarcinoma via machine learning-based Differential Phenotype Immune Score: TPX2 as a key biomarker for immunotherapy resistance

**DOI:** 10.3389/fimmu.2026.1797282

**Published:** 2026-02-27

**Authors:** Xu Zhang, Siyi Sun, Xin Hong, Yi Dong, Xin Wang, Yifan Ma, Kaisheng Yuan, Man Dou, Ying Cao, Xufeng Zhang, Ying Xing

**Affiliations:** 1 The Fourth Department of Medical Oncology, Harbin Medical University Cancer Hospital, Harbin, China; 2 Department of Nutrition and Food Hygiene, School of Public Health, Key Laboratory of Precision Nutrition and Health, Ministry of Education, Harbin Medical University, Harbin, China; 3 Department of Pharmaceutical Sciences, College of Pharmacy and Pharmaceutical Sciences, Washington State University, Spokane, WA, United States

**Keywords:** immune heterogeneity, immunotherapy response, lung adenocarcinoma, single-cell transcriptomics, TPX2

## Abstract

**Background:**

Immune heterogeneity is a major determinant of clinical outcome and immunotherapy responsiveness in lung adenocarcinoma (LUAD). However, the tumor-intrinsic transcriptional programs that drive immune divergence across patients remain insufficiently characterized.

**Methods:**

We constructed an integrated immune landscape of LUAD by combining bulk transcriptomic data, multi-omics profiling, and a large-scale single-cell atlas of non–small cell lung cancer. Immune subtypes were identified through integrative clustering approaches. A machine learning–derived Differential Phenotype Immune Score (DPIS) was developed to quantify immune-related phenotypic variation. Single-cell mapping, regulatory network inference, pan-cancer analyses, protein-level validation, and functional assays were conducted to interrogate key molecular drivers.

**Results:**

Three recurrent immune states were identified, including the Wound Healing, IFN-γ Dominant, and Inflammatory subtypes, each exhibiting distinct immune compositions, metabolic features, signalling activities, and clinical trajectories. Although tumors classified as IFN-γ Dominant or Inflammatory showed comparable sensitivity to immune checkpoint blockade, their baseline prognoses differed substantially, suggesting that immune activation alone does not fully explain outcome heterogeneity. DPIS consistently stratified overall survival across six independent cohorts and was predominantly localized to highly proliferative malignant cells at single-cell resolution. Regulatory network analysis revealed that DPIS-high tumors were governed by cell cycle–associated transcriptional programs. Among the DPIS components, TPX2 emerged as a central regulator linking proliferative signalling to immune suppression, characterized by impaired antigen presentation, reduced immune cell infiltration, and unfavorable immunotherapy responses. Functional experiments further demonstrated that TPX2 promotes tumor cell proliferation, migration, and resistance to apoptosis.

**Conclusion:**

This study identifies a proliferation-driven immune suppression program in LUAD, establishes DPIS as a robust and clinically applicable framework for immune stratification, and highlights TPX2 as a potential therapeutic target for overcoming immune resistance.

## 1 Introduction

Lung adenocarcinoma (LUAD), the most common histological subtype of non–small cell lung cancer (NSCLC), accounts for approximately 40% of all lung cancer cases, with its incidence and mortality rates continuing to rise globally (1). Although the advent of targeted therapies and immune checkpoint inhibitors (ICIs) has markedly improved outcomes for a subset of patients, the overall survival rate remains unsatisfactory. This limited efficacy largely stems from the pronounced heterogeneity of the tumor immune microenvironment (TIME) and the highly variable responses to immunotherapy. Therefore, elucidating the molecular basis of immune heterogeneity in LUAD and developing accurate models to estimate immunotherapy response are of considerable clinical and biological importance (2).

Recent advances in multi-omics integration and single-cell sequencing technologies have provided unprecedented resolution for dissecting the immune ecosystem of tumors (3). Previous studies have demonstrated that LUAD can be stratified into molecular subtypes characterized by distinct immune infiltration patterns, metabolic states, and signaling pathway activation profiles (3–5). These subtypes exhibit divergent immune-escape mechanisms and sensitivities to immune checkpoint blockade (6). However, current classification frameworks remain largely dependent on limited immune signatures or single-omics data, lacking comprehensive cross-platform validation and functional integration. Moreover, the key immune-associated driver genes governing these heterogeneous immune phenotypes remain poorly defined (7, 8).

Targeting Protein for Xklp2 (TPX2), a critical regulator of mitotic spindle assembly, has recently been implicated not only in tumor cell proliferation and division but also in shaping the immune microenvironment through modulation of cell-cycle–associated and stress-response signaling pathways. Nonetheless, its precise role in the formation of LUAD immune heterogeneity and its potential clinical relevance have not been systematically elucidated.

To address these gaps, we performed an integrative analysis combining bulk and single-cell transcriptomic data, machine learning algorithms, and multi-omics profiling to construct a comprehensive immune molecular landscape of LUAD. By classifying and validating LUAD immune subtypes across The Cancer Genome Atlas (TCGA) and multiple independent GEO cohorts, we identified three predominant and biologically distinct immune subtypes—Wound Healing, IFN-γ Dominant, and Inflammatory—each exhibiting unique immune ecosystems, signaling activities, and clinical outcomes. Further multi-layer analyses, including pathway enrichment, metabolic flux inference, and transcription factor activity estimation, delineated functional trajectories underlying these subtypes. We subsequently developed a machine learning–based model termed the Differential Phenotype Immune Score (DPIS), which enabled precise prognostic stratification and demonstrated robust predictive performance across cohorts. Single-cell transcriptomic validation localized DPIS activity to highly proliferative and malignant tumor-cell populations and uncovered their underlying regulatory networks. Finally, we identified TPX2 as a core molecular determinant that is broadly associated with unfavorable prognosis across multiple cancers and linked to an immune-cold, suppressive state—suggesting its potential role as a novel biomarker of immune resistance.

In summary, through the integration of multi-omics data, single-cell validation, and machine learning approaches, this study systematically delineates the immune heterogeneity landscape of LUAD and reveals the mechanistic contribution of the key regulator TPX2. Our findings deepen the understanding of LUAD immunobiology and provide a molecular foundation for precision estimation of immunotherapy response and patient stratification.

## 2 Materials and methods

### 2.1 Data collection and transcriptomic resources

In this study, we systematically collected publicly available transcriptomic resources, encompassing both bulk and single-cell RNA sequencing datasets. Bulk RNA-seq profiles and corresponding clinical annotations for lung adenocarcinoma (LUAD) were obtained from The Cancer Genome Atlas (TCGA-LUAD), while pan-cancer gene expression profiles together with survival information were retrieved from the TCGA Pan-Cancer Atlas via the UCSC Xena platform. In addition, seven independent external cohorts were collected from the Gene Expression Omnibus, including GSE13213 (9), GSE50081 (10), GSE30219 (11), GSE42127 (12), GSE207322 (13), GSE126044 (14), and GSE135222 (15). To further evaluate immune-related features and immunotherapy response, the IMvigor210 cohort was also incorporated (16).

For bulk transcriptomic preprocessing and quality control, all cohorts were analyzed on a TPM scale. GEO expression matrices were retrieved using the GEOquery R package, and TPM values were uniformly transformed using log(x+1) prior to downstream analyses. Gene identifiers were harmonized to official gene symbols, and only genes shared across all cohorts (intersection gene set) were retained to ensure cross-cohort comparability. At the sample level, we removed duplicated entries where applicable and excluded samples lacking essential clinical annotations required for downstream analyses (e.g., survival time/status and key clinicopathological variables). Each cohort was processed independently to avoid cross-study artifacts; when integrated analyses required combining cohorts, batch effects were corrected using the ComBat algorithm with the cohort/study identifier specified as the batch variable, and QC diagnostics (e.g., distribution checks and PCA-based inspection) were performed before and after correction to confirm mitigation of cohort-driven technical variation.

For single-cell transcriptomic analyses, we leveraged a previously curated and integrated non-small cell lung cancer (NSCLC) scRNA-seq resource rather than reprocessing raw sequencing files in-house. This integrated dataset aggregates six GEO cohorts, including GSE131907 (17), GSE136246 (18), GSE148071 (19), GSE153935 (20), GSE127465 (21), and GSE119911 (22), together with an additional cohort generated by the KU Leuven Laboratory for Functional Epigenetics. Upstream preprocessing steps—covering quality control, normalization, batch effect correction/integration, and cell type annotation—were performed by the original authors and are described in detail in the corresponding Scientific Data publication (23). In the present study, we directly utilized the harmonized gene expression matrix and standardized cell type annotations provided by this resource for downstream analyses. The processed data objects are publicly available via Figshare (collection DOI: https://doi.org/10.6084/m9.figshare.c.6222221.v3) (23).

### 2.2 Identification of immune molecular subtypes

To delineate immune associated molecular subtypes in lung adenocarcinoma, immune subtype classification was performed based on transcriptomic profiles. The transcript per million normalized gene expression matrix derived from the TCGA LUAD cohort was initially curated to include only primary tumor specimens, and duplicated sample identifiers were excluded. Expression values were subsequently transformed using the logarithmic formula log(TPM + 1) to reduce heteroscedasticity and improve data stability. Immune subtype assignment was conducted using the ImmuneSubtypeClassifier R package version 1.1.0, which applies an ensemble learning based classification framework. Gene expression matrices were annotated according to gene symbols, and the quality of gene matching was systematically evaluated using the geneMatch() and geneMatchErrorReport() functions to ensure reliable subtype prediction.

### 2.3 Immune infiltration analysis

Immune infiltration analysis was performed using bulk RNA sequencing data from lung adenocarcinoma samples. The proportions of immune and stromal components in the tumor microenvironment were estimated using the ESTIMATE algorithm version 1.0.13 (24). Before analysis, the gene expression matrix was filtered to retain genes with common expression across samples. Stromal score, immune score, and tumor purity were calculated using the Affymetrix platform option. All calculated scores were standardized prior to subsequent analyses. The relative abundance of immune and stromal cell populations was assessed using gene set variation analysis version 1.46.0 (25). A curated gene signature set representing twenty four immune and stromal cell types was used, including T cell subsets, B cells, macrophages, and dendritic cells. GSVA was applied to compute enrichment scores for each cell type on a per sample basis. Expression levels of selected immune checkpoint genes were extracted from normalized transcriptomic data, including PDCD1, CD274, PDCD1LG2, CTLA4, TNFRSF9, and TNFRSF4. In addition, T cell related functional programs were quantified using the TCellSI framework version 0.1.0 (26), which calculates sample level scores for predefined T cell states such as exhaustion, cytotoxicity, and activation. All derived immune related metrics were subsequently used for downstream analyses in combination with immune subtype classifications.

### 2.4 Construction of a prognostic signature using machine learning approaches

A prognostic gene signature associated with immune subtypes in lung adenocarcinoma was constructed using a sequential analytical workflow integrating differential expression analysis, survival analysis, and machine learning–based feature selection. Differentially expressed genes were identified between the IFN-γ dominant subtype and the inflammatory subtype within the TCGA-LUAD cohort using limma (v3.52.4) (27). Genes with an absolute log2 fold change > 1.5 and an adjusted P value < 0.01 were retained. Candidate prognostic genes were then screened by univariate Cox proportional hazards regression using overall survival (OS), and genes with P < 0.05 were forwarded to model development. The TCGA cohort served as the training dataset, while five independent GEO cohorts (GSE13213, GSE50081, GSE30219, GSE31210, and GSE42127) were used for external validation. Expression matrices and survival annotations were harmonized across cohorts; only genes consistently present across all datasets were retained.

Prognostic model development was implemented using the open-source machine-learning framework Mime (loaded as Mime1) (28), which is designed for multi-cohort transcriptomic modeling and provides integrated procedures for survival modeling, feature selection, and performance visualization.he core function ML.Dev.Prog.Sig was used to construct prognosis models from a training cohort and multiple validation cohorts, where the input matrix follows the recommended format (sample identifier, OS time, and OS status as the first columns, followed by gene expression values scaled as log2(x+1)). Within ML.Dev.Prog.Sig, optional univariate Cox filtering can be applied to candidate genes in the training cohort (unicox.filter.for.candi with a configurable unicox_p_cutoff), and three analysis modes are available (all, single, and double), with all evaluating ten built-in algorithms and their combinations. In this study, we used the Mime1 framework to evaluate multiple modeling strategies, including stepwise Cox regression and elastic-net regularization, and selected the final signature based on cross-cohort performance stability (Harrell’s C-index) and time-dependent ROC/AUC at 1, 3, and 5 years. Furthermore, a meta-analysis of univariate Cox results across all cohorts was conducted to prioritize genes with consistent prognostic effects, following the package-supported workflow for univariate Cox summarization and meta-analysis. For single-cell analyses, we did not re-train DPIS as a prognostic model at the cellular level. Instead, to characterize the cell-type distribution of the DPIS transcriptional program, we computed a DPIS-10 gene-set activity score using the same 10 genes in the bulk DPIS model (TPX2, UBE2C, CDC20, BIRC5, SCGB3A1, SFTPB, CACNA2D2, CYP4B1, MYBL2, and SUSD2). This single-cell score reflects relative expression activity of the DPIS gene set (module score) and does not apply the bulk Cox coefficients.

**Figure 1:**
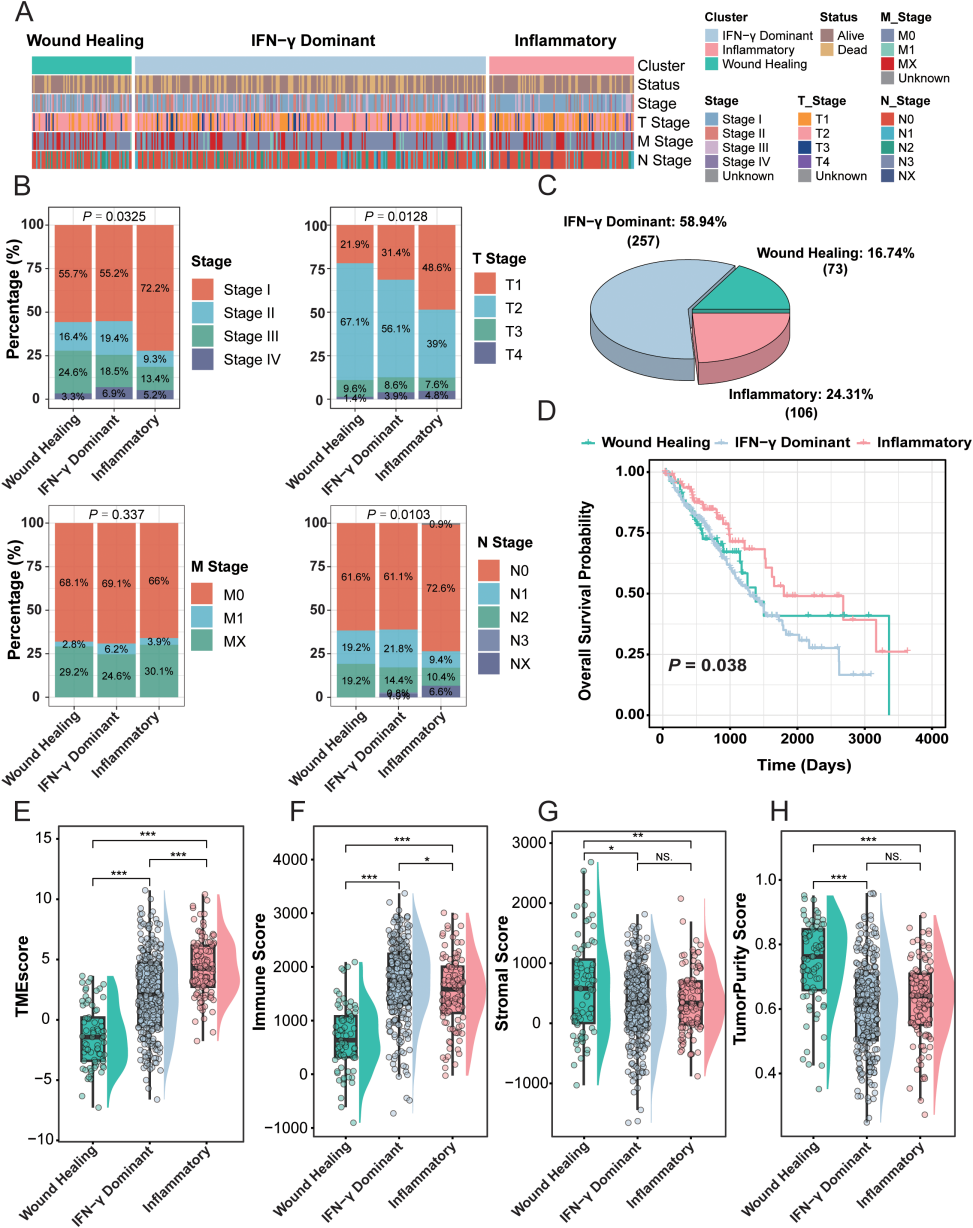
Identification and characterization of LUAD immune subtypes. **(A)** Heatmap showing the three representative immune subtypes—Wound Healing, IFN-γ Dominant, and Inflammatory—identified by unsupervised clustering of immune-related gene expression profiles in LUAD. Clinical annotations (survival status, stage, and T/M/N classification) are displayed beneath the heatmap. **(B)** Distribution of the three immune subtypes across major clinicopathological parameters, including clinical stage, T stage, M stage, and N stage. **(C)** Proportions of each immune subtype within the LUAD cohort. **(D)** Kaplan–Meier survival curves comparing overall survival among the three immune subtypes. **(E–H)** Comparison of tumor microenvironment characteristics among the three immune subtypes, including the TME score, Immune score, Stromal score, and Tumor Purity. *P < 0.05; **P < 0.01; ***P < 0.001. “NS” stands for “not significant” (P ≥ 0.05).

**Figure 2:**
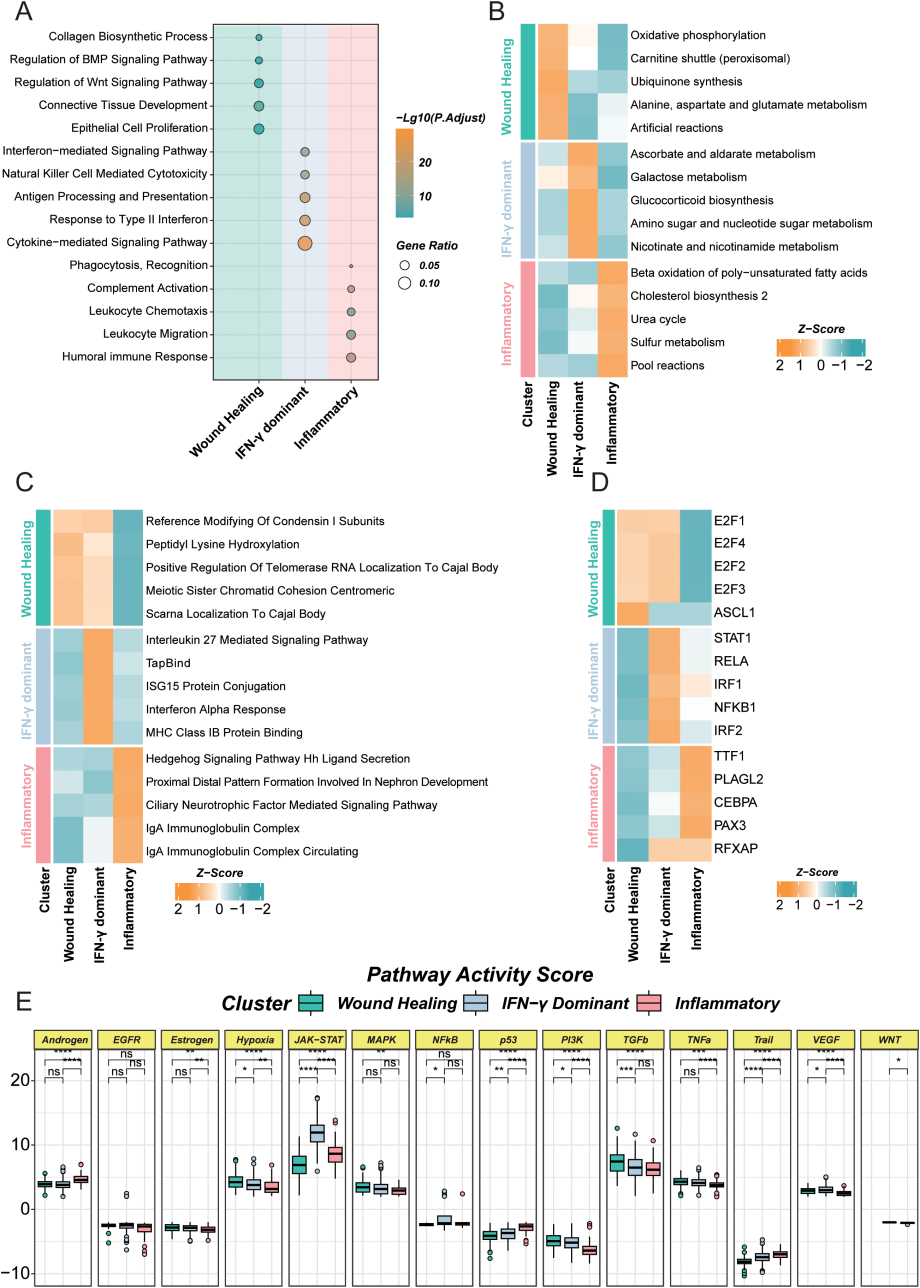
Multidimensional features of functional pathways, metabolic flux, transcription-factor activity, and signaling across LUAD immune subtypes. **(A)** GO Biological Process enrichment based on subtype-specific DEGs, showing representative categories and adjusted significance for Wound Healing, IFN-γ Dominant, and Inflammatory subtypes. **(B)** Heatmap of GSVA scores from integrated gene-set collections (Hallmarks, KEGG, GO BP/CC/MF, Reactome), displaying relative activity (Z-score) of selected pathways across subtypes. **(C)** Heatmap of METAFlux-inferred metabolic pathway activities (representative entries per subtype), showing relative activity (Z-score) across metabolic subsystems. **(D)** Heatmap of transcription-factor activities computed with decoupleR (top variable TFs), summarizing subtype-specific regulatory programs (Z-score). **(E)** Faceted boxplots of canonical signaling pathway activities (e.g., JAK–STAT, MAPK, PI3K, p53, NF-κB); asterisks denote significance levels for pairwise comparisons. *P < 0.05; **P < 0.01; ***P < 0.001; ****P < 0.0001. “ns” stands for “not significant” (P ≥ 0.05).

**Figure 3:**
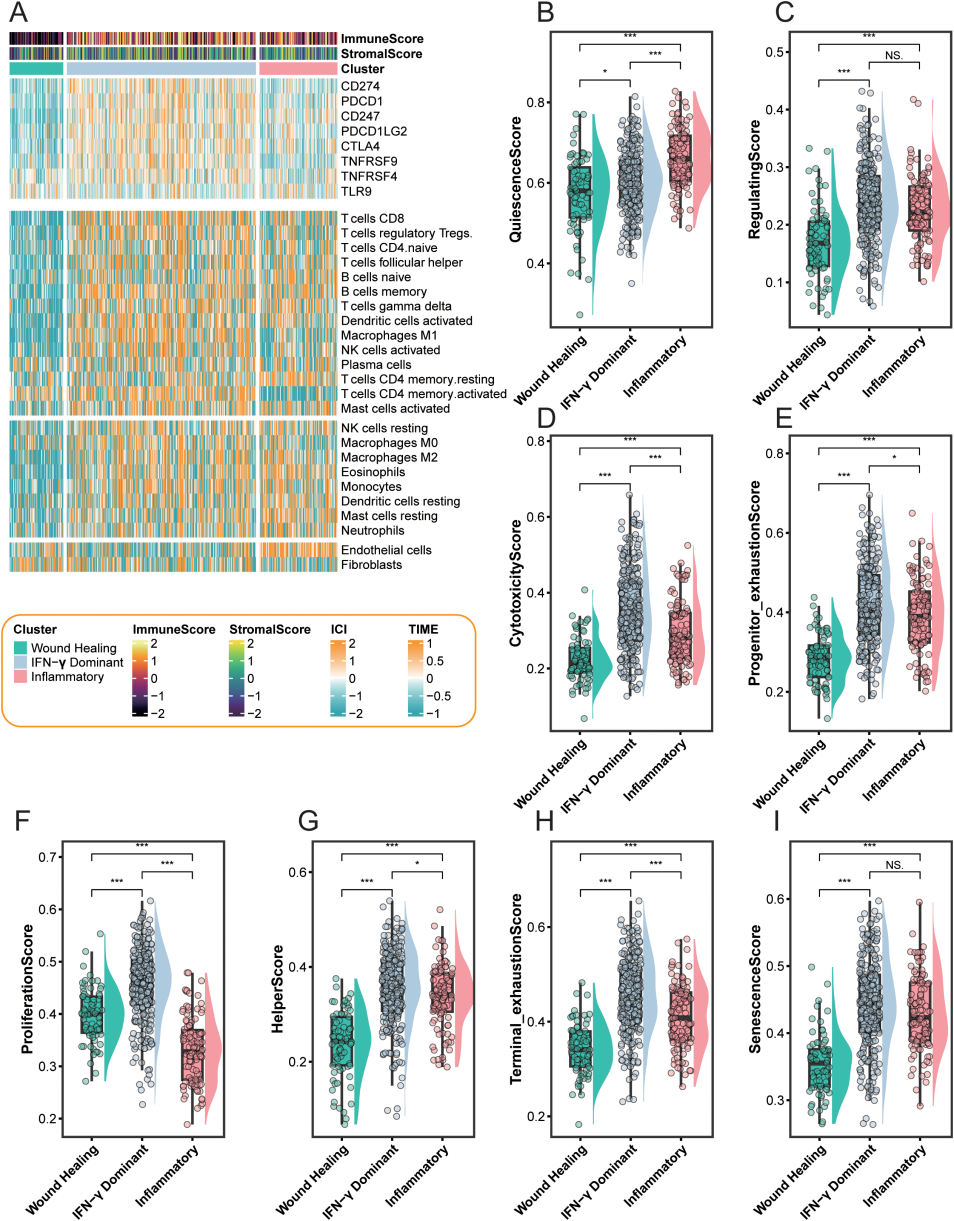
Immune checkpoint expression, immune cell infiltration, and T-cell functional states across LUAD immune subtypes. Heatmap showing the expression patterns of immune checkpoint genes (CD274, PDCD1, CTLA4, TNFRSF9, TNFRSF4, TLR9) and enrichment scores of immune cell populations among the three immune subtypes—Wound Healing, IFN-γ Dominant, and Inflammatory. **(A)** Heatmap summarizing ImmuneScore and StromalScore, immune-subtype (cluster) assignment (Wound Healing, IFN-γ Dominant, and Inflammatory), the expression of selected immune checkpoint–related genes (CD274, PDCD1, CD247, PDCD1LG2, CTLA4, TNFRSF9, TNFRSF4, and TLR9), and the estimated abundances of immune/stromal cell populations across individual LUAD samples. Color scales represent scaled values as indicated in the legend. ImmuneScore and StromalScore annotations are displayed on top. **(B–I)** Comparison of T-cell functional scores among the three immune subtypes, including QuiescenceScore **(B)**, RegulatingScore **(C)**, CytotoxicityScore **(D)**, Progenitor/exhaustionScore **(E)**, ProliferationScore **(F)**, HelperScore **(G)**, Terminal exhaustionScore **(H)**, and SenescenceScore **(I)**. *P < 0.05; ***P < 0.001. “NS” stands for “not significant” (P ≥ 0.05).

**Figure 4:**
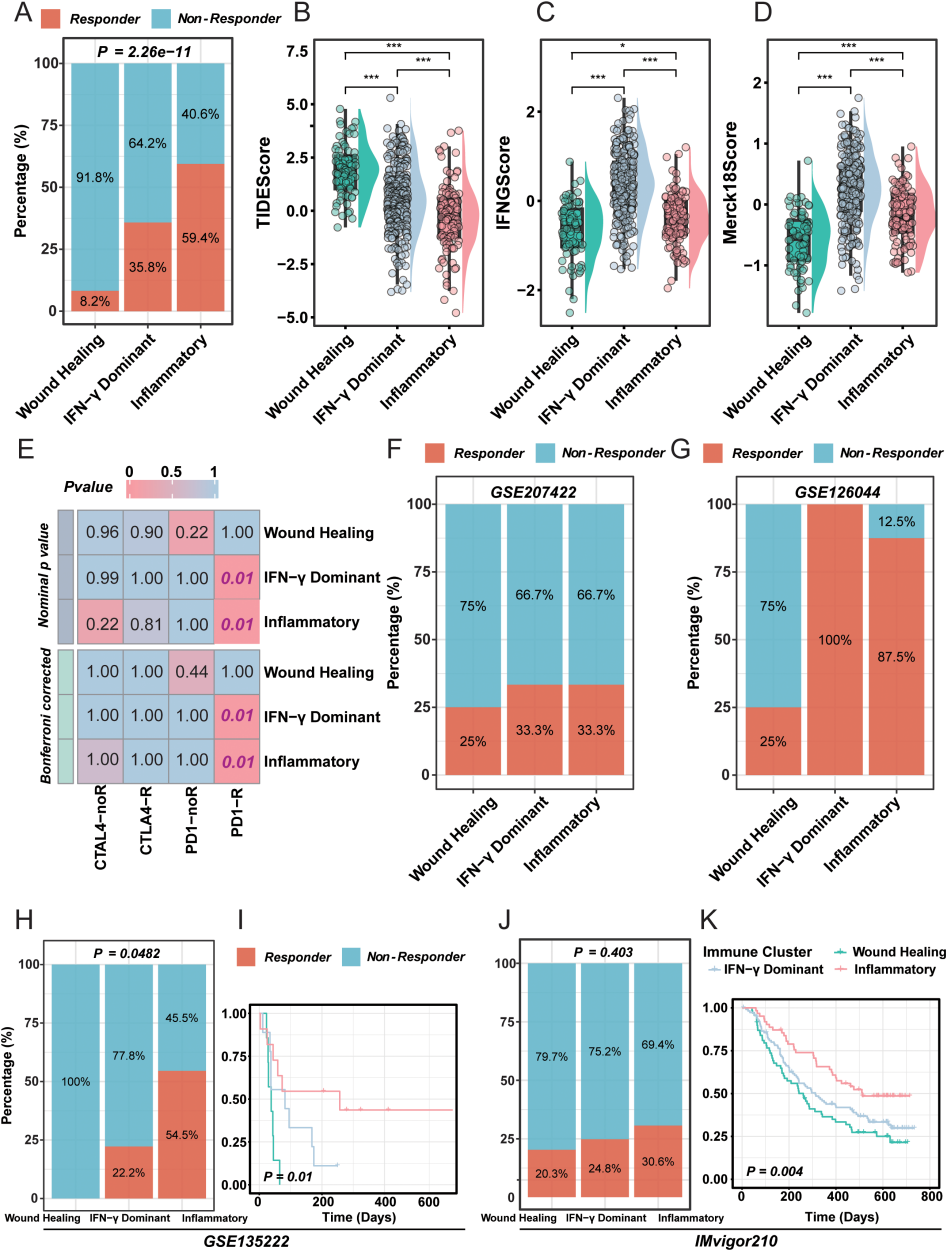
Distinct immunotherapy response landscapes across LUAD immune subtypes. **(A)** Distribution of predicted responders and non-responders to immune checkpoint blockade (ICB) among LUAD immune subtypes based on TIDE prediction. **(B–D)** Comparison of TIDE-derived immunotherapy-related indices (TIDE score, IFNG score, and Merck18 score) across Wound Healing, IFN-γ Dominant, and Inflammatory subtypes. **(E)** SubMap analysis showing transcriptional similarity between LUAD immune subtypes and known ICB responder phenotypes from published melanoma cohorts (CTLA4-R, PD1-R). **(F–K)** External immunotherapy cohort validation (IMvigor210, GSE135222, GSE207422, and GSE126044) illustrating subtype-specific response rates and survival outcomes, demonstrating that Inflammatory and IFN-γ Dominant subtypes exhibit enhanced clinical benefit from ICB, whereas the Wound Healing subtype remains largely refractory. *P < 0.05; ***P < 0.001.

**Figure 5:**
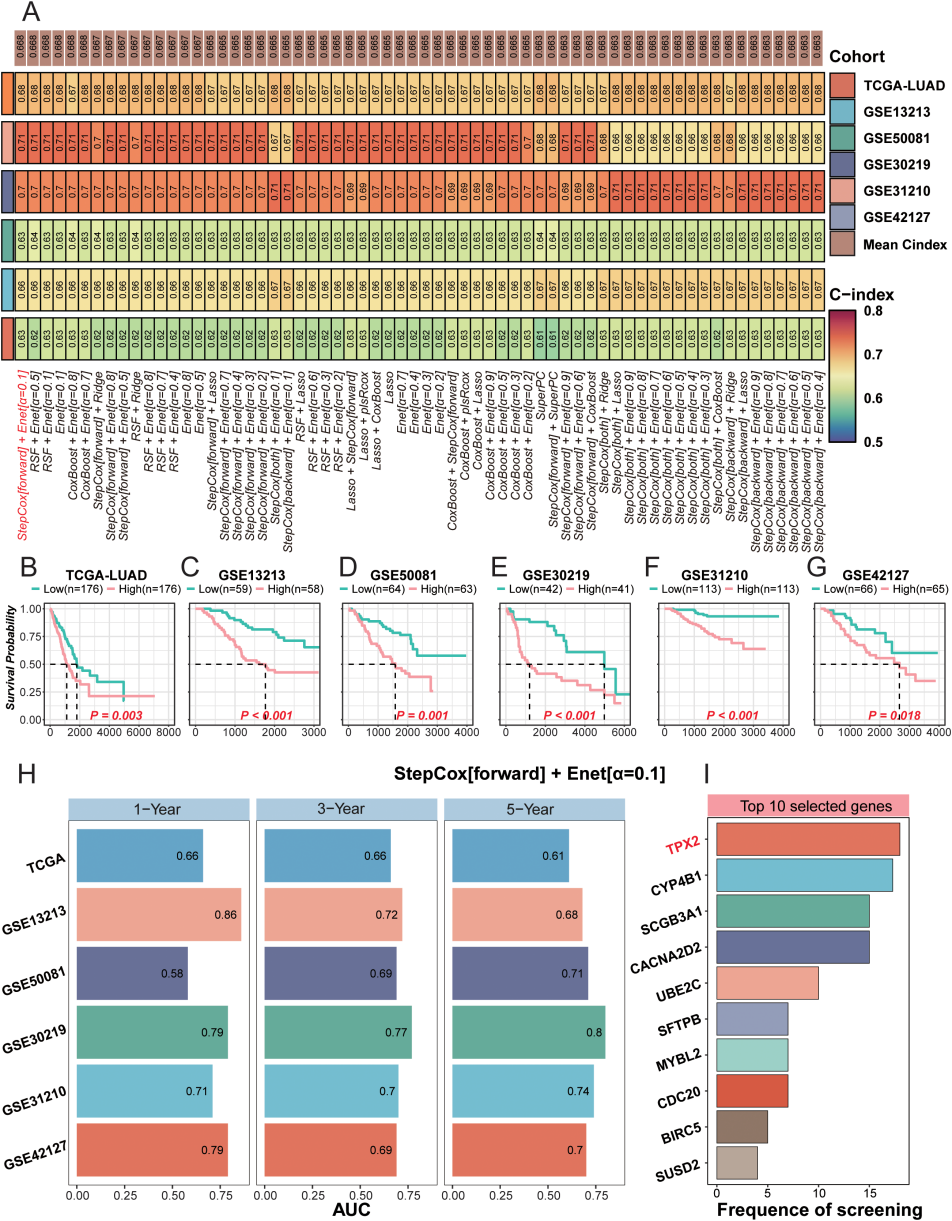
Construction and validation of the DPIS (Differential Phenotype Immune Score) model using the Mime1 framework. **(A)** Heatmap of C-index values across multiple algorithms evaluated in the TCGA-LUAD training cohort and five GEO validation datasets. **(B–G)** Kaplan–Meier overall survival curves comparing high- and low-risk groups defined by the DPIS model across six independent cohorts. **(H)** Left: time-dependent ROC curves summarizing 1-, 3-, and 5-year AUC values for each dataset. Right: the ten most frequently selected genes contributing to DPIS feature construction. **(I)** Bar plot showing the top 10 genes most frequently selected during the feature-screening procedure in the StepCox (forward) + Enet (α = 0.1) modeling pipeline.

**Figure 6:**
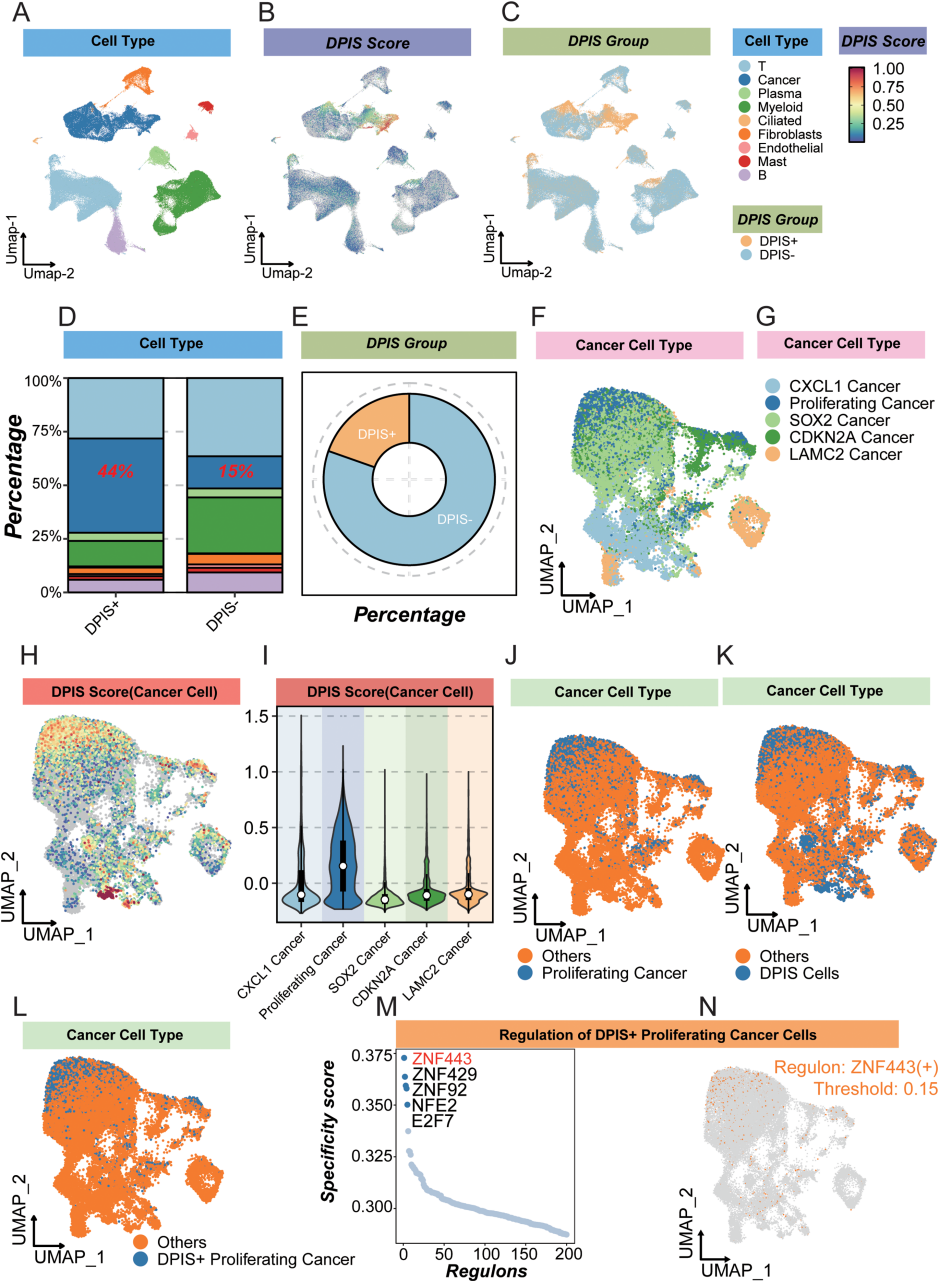
Single-cell mapping of the DPIS signature in LUAD. **(A)** UMAP of all cells colored by major cell types. **(B)** UMAP showing continuous DPIS scores. **(C)** Cells stratified into DPIS^+^ and DPIS^-^ groups. **(D)** Stacked bar plot comparing cell-type compositions between DPIS^+^ and DPIS^-^ cells (percentage annotated). **(E)** Donut chart summarizing the overall fraction of DPIS^+^ vs. DPIS^-^ cells and their cell-type breakdown. **(F)** UMAP of malignant cells annotated by tumor states (CXCL1, Proliferating, SOX2, CDKN2A, LAMC2). **(G)** DPIS score overlaid on malignant-cell UMAP. **(H)** Spatial distribution of DPIS scores within malignant cells. **(I)** Violin/box plots of DPIS scores across malignant states. **(J)** UMAP highlighting proliferating vs. other malignant cells. **(K)** UMAP highlighting DPIS^+^ vs. other malignant cells. **(L)** UMAP highlighting DPIS^+^ Proliferating Cancer cells. **(M)** Ranked regulon specificity scores (RSS) for the DPIS^+^ proliferating population. **(N)** Representative regulon activity map (e.g., ZNF443(+)) projected onto the malignant-cell UMAP.

**Figure 7:**
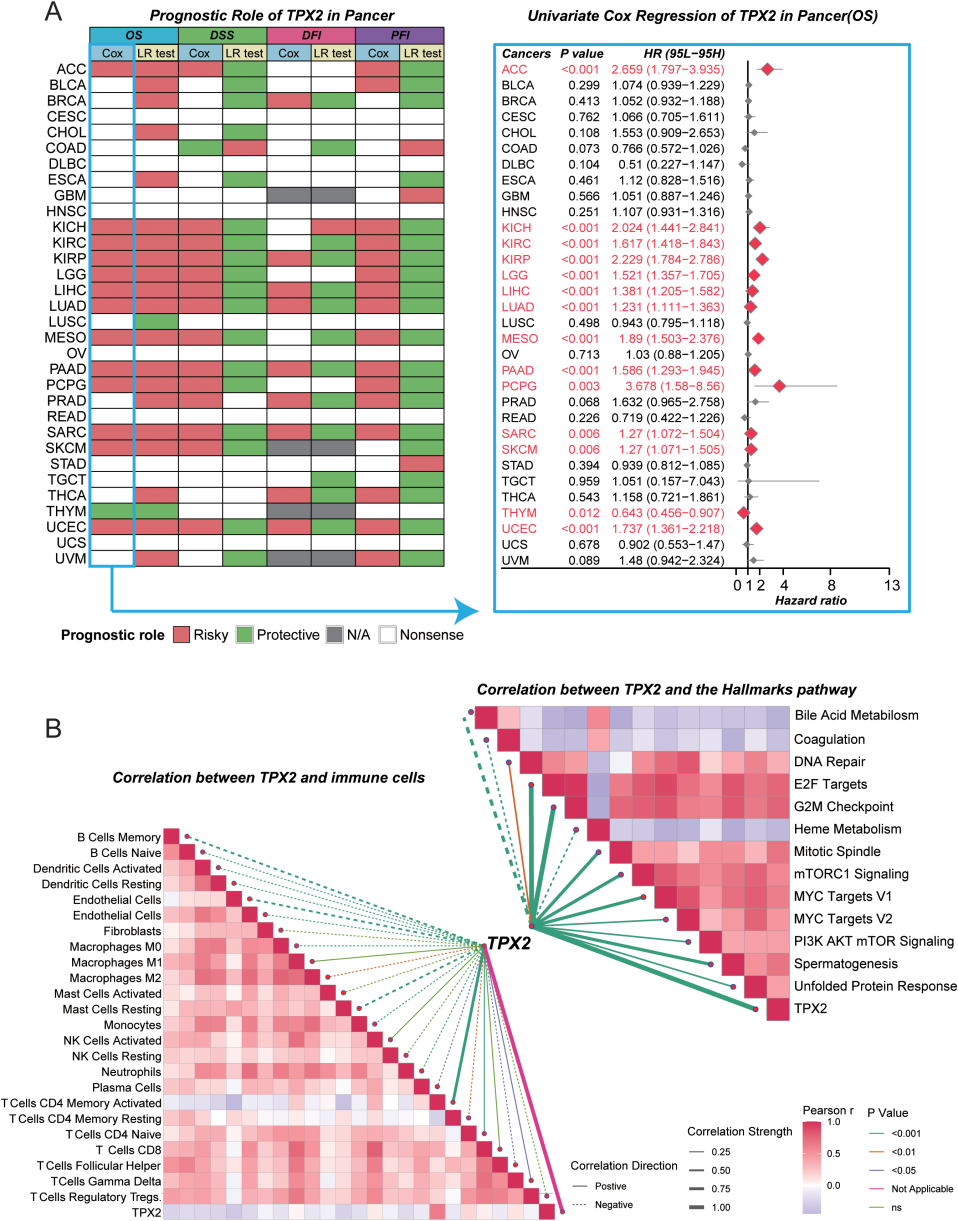
Pan-cancer prognostic and functional characterization of TPX2. **(A)** Prognostic overview of TPX2 across 32 TCGA cancers. Left, heatmap summarizing TPX2’s prognostic roles across OS, DSS, DFI, and PFI using Cox and log-rank tests (red = risky, green = protective, gray = not applicable, white = non-significant). Right, forest plot showing univariate Cox hazard ratios (diamonds) with 95% confidence intervals for overall survival. **(B)** Correlation map of TPX2 in LUAD. Left, pairwise correlations between TPX2 and immune-cell gene signatures, where line color indicates correlation direction and thickness reflects effect size. Right, correlations between TPX2 and Hallmark pathways showing strong positive associations with proliferative signatures and negative correlations with metabolic processes.

To ensure reproducibility, all analysis and figure-generation scripts are publicly available. The Scripts directory contains the analysis and visualization scripts corresponding to **Figures 1**–**7** (including the complete workflow used for **Figure 5**), and the R directory provides auxiliary functions used by these scripts.

### 2.5 Functional analysis of immune subtypes in LUAD

Functional analyses were performed to assess molecular programs associated with immune subtypes in lung adenocarcinoma. Multiple analytical strategies were applied, including functional enrichment analysis, pathway activity estimation, metabolic flux inference, and regulatory network analysis. Gene Ontology enrichment analysis was conducted using the compareCluster function implemented in the clusterProfiler package version 4.8.1 (29). Subtype specific gene sets were generated and enrichment analysis was performed for the biological process category. Redundant Gene Ontology terms were filtered using the simplify function with default parameters. Enrichment results were summarized and prepared for downstream visualization. Pathway activity was evaluated using Gene Set Variation Analysis with gene sets derived from the Hallmark, KEGG, Reactome, and Gene Ontology collections obtained from MSigDB version 2025.1. Enrichment scores were calculated for each sample based on normalized transcriptomic data. Differences in pathway activity across immune subtypes were assessed using linear modeling. Pathway level results were organized and visualized using heatmap based representations. Metabolic pathway activity was inferred using the METAFlux framework version 1.0 (30), which integrates transcriptomic data with a genome scale metabolic model derived from Human GEM. Flux distributions were computed under a predefined nutrient environment corresponding to human blood. Reaction level flux values were aggregated to generate pathway level scores, which were subsequently compared across immune subtypes. Regulatory activity analysis was performed using the decoupleR package version 2.3.1 (31). Signaling pathway activity was inferred using the PROGENy resource, while transcription factor activity was estimated based on regulons obtained from the COLLECTRI database. Both multilinear and univariate linear models were applied to derive activity scores from gene expression data. Resulting pathway and transcription factor activity matrices were used for downstream comparative analyses and visualization.

### 2.6 Association analysis of immune subtypes with immunotherapy outcomes

The association between immune subtypes and immunotherapy related outcomes in lung adenocarcinoma was evaluated using a combination of computational prediction methods and independent clinical immunotherapy cohorts. The Tumor Immune Dysfunction and Exclusion framework was applied to transcriptomic data from the TCGA LUAD cohort to calculate immune dysfunction scores, immune exclusion scores, and interferon gamma related signatures (32). Subtype level transcriptional similarity to established immunotherapy response profiles was examined using SubMap analysis (33). Expression profiles derived from lung adenocarcinoma immune subtypes were compared with reference melanoma cohorts treated with immune checkpoint blockade targeting programmed cell death protein one or cytotoxic T lymphocyte associated protein four. External validation analyses were conducted using five independent non-small cell lung cancer cohorts treated with immune checkpoint inhibitors, including IMvigor210, GSE135222, GSE207322, GSE207422, and GSE126044. Immune subtypes within each cohort were assigned using expression based classification approaches consistent with those applied in the discovery dataset. Associations between immune subtype and therapeutic response categories, defined according to RECIST criteria or best overall response, were evaluated using Fishers exact test. Time to event outcomes were analyzed in cohorts with available survival information. Overall survival and progression free survival were assessed using Kaplan Meier estimation and Cox proportional hazards regression models, as appropriate. All statistical analyses were conducted using standard R based survival analysis workflows.

### 2.7 Single cell transcriptomic analysis and transcriptional regulatory network inference

Single cell transcriptomic analysis was conducted using a previously curated and integrated dataset comprising multiple non-small cell lung cancer cohorts. Standard preprocessing procedures were applied to the single cell RNA sequencing data using the Seurat package version 4.3.0, including data normalization, feature scaling, and dimensionality reduction. Cell identity annotations were assigned based on harmonized metadata provided with the integrated dataset. Immune related transcriptional patterns at the single cell level were quantified using a scoring framework termed Differential Phenotype of Immune Signature. This score was calculated based on the expression of selected immune associated and proliferation related genes. Subsequent analyses were restricted to malignant epithelial cell populations, with a focus on proliferating tumor cells. To infer transcriptional regulatory networks associated with Differential Phenotype of Immune Signature variation, a gene regulatory network analysis pipeline was implemented using pySCENIC. The workflow included construction of meta cells, transcription factor target gene inference using the GRNBoost2 algorithm, motif enrichment analysis using cisTarget, and quantification of regulon activity at the single cell level using AUCell. Regulon activity scores were computed across malignant cell populations for downstream comparative analyses. Batch effect correction and dataset integration were performed using Harmony version 0.1.1. All analyses were conducted using Seurat version 4.3.0 (34), SCENIC version 1.2.0 (35), Harmony version 0.1.1 (36), and pySCENIC version 0.12.1 (35). CisTarget databases and motif annotations corresponding to the human genome build hg38 were obtained from https://resources.aertslab.org/cistarget/databases/ and applied using default parameters.

### 2.8 Public protein level evidence from the human protein atlas

Protein level information for TPX2 was obtained from publicly available resources provided by the Human Protein Atlas database. Immunohistochemistry staining data derived from normal tissues and tumor tissues were retrieved to enable protein expression assessment across tissue types. These data were used as an independent protein level reference corresponding to transcriptomic findings. In addition to tissue based immunohistochemistry data, confocal immunofluorescence images generated from human cell lines were collected from the same database. These images were used to examine the subcellular distribution of TPX2. All protein related data were accessed through the Human Protein Atlas portal at https://www.proteinatlas.org and processed according to the database annotations and documentation.

### 2.9 Cell lines and culture conditions

Human lung cancer cell lines H1299, 95D, A549, and H1650 were used in this study, together with the normal human bronchial epithelial cell line HBE. All cell lines were obtained from the laboratory cell bank and maintained according to standard cell culture procedures. Cells were cultured in Dulbeccos Modified Eagle Medium supplied by Gibco and supplemented with 10 percent fetal bovine serum and 1 percent penicillin streptomycin. Cell cultures were maintained at 37 degrees Celsius in a humidified incubator with 5 percent carbon dioxide.

### 2.10 Transient transfection procedure

Cells were seeded into six well culture plates at a density of two hundred thousand cells per well and incubated for twenty four hours to allow cell attachment. After incubation, a transfection mixture was prepared consisting of two hundred microliters of buffer, four microliters of Polyplus jetPRIME reagent, and five microliters of small interfering RNA. The transfection mixture was added directly to each well following the manufacturer recommended protocol. Cells were incubated with the transfection complexes for six hours. After this incubation period, the transfection medium was removed and replaced with fresh complete culture medium. Cells were then maintained under standard culture conditions for subsequent experiments.

### 2.11 Protein extraction and Western blotting

When cell cultures reached approximately eighty to ninety percent confluence, total cellular proteins were extracted using a commercial protein extraction kit from Biyuntian. Cells were collected by centrifugation and lysed in RIPA buffer P0013B obtained from Biyuntian China. Lysis was performed on ice for thirty minutes with gentle mixing at ten minute intervals. Cell lysates were subsequently clarified by centrifugation at twelve thousand times gravity for fifteen minutes, and the supernatants were collected for further analysis. Protein samples were mixed with SDS PAGE loading buffer P0015 from Biyuntian China and heated at ninety five degrees Celsius for ten minutes to achieve protein denaturation. Denatured proteins were separated by polyacrylamide gel electrophoresis and transferred onto polyvinylidene fluoride membranes. Membranes were blocked using non-fat milk solution for two hours at room temperature and then incubated overnight at four degrees Celsius with primary antibodies targeting TPX2 and GAPDH. After washing three times with Tris buffered saline containing Tween, membranes were incubated with the appropriate secondary antibodies for one hour at room temperature. Membranes were washed again and protein signals were detected according to standard protocols. All experiments were independently repeated at least three times.

### 2.12 Cell counting Kit 8 based cell proliferation assay

Cells were seeded into ninety six well culture plates at a density of five thousand cells per well. After cell seeding, cultures were maintained under standard conditions. At predefined time points of twenty four hours, forty eight hours, and seventy two hours, Cell Counting Kit 8 reagent was added to each well according to the manufacturer instructions. Plates were then incubated for two hours at thirty seven degrees Celsius. Following incubation, absorbance values were measured at a wavelength of four hundred fifty nanometers using a microplate reader. The obtained optical density values were collected for subsequent analysis. Each experimental condition was assessed using at least three technical replicates to ensure reproducibility.

### 2.13 Wound healing assay

Cells were seeded into six-well plates and grown to approximately 90–100% confluence. A sterile 10-μL pipette tip was used to create a straight scratch across the cell monolayer. The wells were gently rinsed with PBS to remove detached cells, followed by the addition of serum-free medium. Images of the wound area were captured at 0 and 24 hours using an inverted microscope. Wound closure was quantified by measuring the remaining scratch area at each time point. All experiments were performed in three independent replicates to ensure the robustness of the results.

### 2.14 Cell apoptosis detection

Adherent lung adenocarcinoma cells were washed and detached using trypsin solution C0205 obtained from Biyuntian Biotechnology. Collected cells were counted, and cell suspensions were adjusted to a final density of one million cells per milliliter. Apoptosis analysis was performed using an Annexin V FITC and propidium iodide staining kit FXP018–100 provided by Beijing Four A Biotech. Stained cells were analyzed by flow cytometry according to the manufacturer recommended procedure. Fluorescence signals were recorded for subsequent analysis. Each experiment was conducted using independent biological replicates.

### 2.15 Statistical analysis

All statistical analyses were performed using the R software environment version 4.2.2. Associations between variables and survival outcomes were evaluated using univariate Cox proportional hazards regression models and Kaplan Meier survival analysis. Correlation analyses were conducted using the Pearson correlation method. Gene set variation analysis was applied for pathway level activity estimation. Unless otherwise specified, statistical significance was determined using a two sided P value threshold of less than 0.05.

## 3 Results

### 3.1 Identification of immune subtypes reveals clinical heterogeneity and distinct tumor ecosystems in LUAD

To systematically dissect immune heterogeneity in lung adenocarcinoma (LUAD), we assigned tumor immune subtypes using the ImmuneSubtypeClassifier framework, which classifies samples into six established pan cancer immune classes based on immune related gene expression patterns (485 signature genes; XGBoost classifier). To ensure robust subtype calls, only samples with a maximum class confidence score greater than 0.6 were retained for downstream analyses. Under this stringent criterion, the resulting six class distribution in TCGA LUAD was Wound Healing (n = 73), IFNγ Dominant (n = 258), Inflammatory (n = 106), Lymphocyte Depleted (n = 8), Immunologically Quiet (n = 0), and TGFβ Dominant (n = 0) (full distribution shown in **Supplementary Data 1**). Given that three classes accounted for 98.2 percent of robustly classified cases (437 of 445) and provided adequate statistical power for subtype wise comparisons, the subsequent analyses focused on the three predominant and representative subtypes, namely Wound Healing, IFNγ Dominant, and Inflammatory. As shown in the heatmap (**Figure 1A**), the three predominant immune phenotypes exhibited clear stratification across multiple clinical variables, including survival status, overall stage, and T, N, and M categories, highlighting substantial clinical and biological heterogeneity. Within this predominant subset (n = 437), IFN-γ Dominant accounted for 58.9% (258/437), Inflammatory for 24.3% (106/437), and Wound Healing for 16.7% (73/437) (**Figure 1C**).

Clinicopathological comparisons further showed marked differences in disease progression across subtypes (**Figure 1B**). The IFN-γ Dominant subtype was enriched in advanced-stage tumors (stage III–IV, P = 0.0325), showed a higher proportion of T3–T4 lesions (P = 0.0128), and exhibited increased frequencies of N2–N3 nodal involvement (P = 0.0103), consistent with a more aggressive clinical presentation. In contrast, the Inflammatory subtype was overrepresented in early-stage disease (stage I–II), consistent with a relatively less advanced phenotype at diagnosis.

Consistent with these clinicopathological patterns, overall survival differed significantly among the three subtypes (**Figures 1D**, P = 0.038). Patients classified as Inflammatory demonstrated the most favorable survival, whereas those in the IFN-γ Dominant and Wound Healing subtypes had comparatively poorer outcomes. To account for potential confounding from baseline clinicopathological imbalances (e.g., stage and T/N/M categories), we additionally performed a multivariable Cox proportional hazards analysis in the TCGA-LUAD cohort. After adjustment for sex, overall stage, and detailed T/N/M categories, DPIS remained an independent risk factor for overall survival (HR = 3.65, 95% CI 1.83–7.25, P = 2.26×10^-4^; **Supplementary Data S4**), supporting that DPIS provides prognostic information beyond standard clinical variables.

To further characterize the immunological basis underlying these differences, we compared tumor microenvironment (TME) features across subtypes (**Figures 1E–H**). The Inflammatory subtype showed significantly higher TME and Immune scores (all P < 0.001), consistent with enhanced immune and stromal components. By contrast, the Wound Healing subtype exhibited the highest tumor purity (P < 0.001) and the lowest immune infiltration, consistent with an immune-excluded or immune-suppressive microenvironment, which may partly explain its unfavorable prognosis.

### 3.2 Multilayer characterization of LUAD immune subtypes across pathways, metabolic flux, transcription factors, and signaling activities

To delineate functional divergence among LUAD immune subtypes, we integrated multi-layer evidence from **Figures 2A–E**, encompassing GO biological processes, broad gene-set activity, metabolic flux, transcription-factor activity, and canonical pathway scores. The Wound Healing subtype was enriched for collagen biosynthesis, connective-tissue development and Wnt/BMP regulation, accompanied by higher oxidative phosphorylation, carnitine shuttle and amino-acid metabolism, prominent activity of proliferation/epithelial lineage factors (E2F, ASCL1, TTF1) and elevated TGF-β/WNT/EGFR signaling—collectively defining a tissue-repair/stroma-remodeling program. The IFN-γ Dominant subtype showed pronounced enrichment of type-II interferon and cytokine signaling, antigen processing/presentation and NK-cell cytotoxicity, together with gene-set and flux patterns consistent with immunoresponsive metabolic re-programming (carbohydrate/nucleotide-sugar and NAD metabolism and steroidogenesis); this was paralleled by activation of the STAT1/IRF axis (with RELA/NFKB1) and higher activities of JAK–STAT, MAPK, PI3K, p53 and hypoxia pathways, delineating an antigen-driven, highly activated state coupled to stress/growth signaling. The Inflammatory subtype was characterized by enrichment of complement and humoral immune responses, leukocyte chemotaxis/migration and phagocytic recognition, alongside lipid β-oxidation, cholesterol biosynthesis and nitrogen/sulfur metabolism, dominant NF-κB/CEBPA programs, and increased NF-κB/TNFα (with partial VEGF/WNT) signaling. Together, **Figure 2** resolves a continuum across subtypes—from stroma-remodeling/repair (Wound Healing) through interferon-driven hyperactivation (IFN-γ Dominant) to inflammation- and humoral-immunity–dominated states (Inflammatory)—providing mechanistic context for their divergent tumor ecosystems.

### 3.3 Immune checkpoint landscape and T-cell functional profiling reveal distinct immune ecologies among LUAD subtypes

To gain deeper insight into the immune landscape of LUAD, we comprehensively analyzed immune checkpoint expression, immune cell composition, and T-cell functional states (**Figures 3A–I**). The integrative heatmap (**Figure 3A**) revealed marked differences among the three immune subtypes in both immune checkpoint gene expression (e.g., CD274, PDCD1, CTLA4, TNFRSF9) and immune cell infiltration patterns. Both the IFN-γ Dominant and Inflammatory subtypes exhibited elevated immune and stromal scores, yet they represented fundamentally distinct immune phenotypes. The IFN-γ Dominant subtype was characterized by pronounced upregulation of immune checkpoint molecules, heightened T-cell activation, and increased exhaustion-associated signatures, indicating a hyper-responsive but concurrently suppressive immune state. In contrast, the Inflammatory subtype was dominated by broad immune cell enrichment and robust inflammatory activity, reflecting a more canonical immune-activated microenvironment. By comparison, the Wound Healing subtype displayed relatively low levels of immune infiltration but significant enrichment of endothelial and fibroblast populations, suggesting a tissue-repairing and stromal-remodeling phenotype accompanied by immune quiescence. Further analysis of T-cell functional states (**Figures 3B–I**) revealed clear stratification in immune activation hierarchies across subtypes. The IFN-γ Dominant subtype exhibited the highest scores across multiple dimensions—including cytotoxicity, proliferation, helper activation, and exhaustion—consistent with a highly activated and antigen-driven T-cell program. The Inflammatory subtype maintained intermediate but balanced immune activity, whereas the Wound Healing subtype showed the lowest T-cell functionality, consistent with its immunologically inert state.

### 3.4 Enhanced clinical benefit of inflammatory and IFN-γ dominant immune subtypes under immunotherapy

Within the TCGA-LUAD cohort, the predicted immunotherapy response rates varied markedly among the three immune subtypes (**Figure 4A**; χ² P = 2.26 × 10^-^¹¹). The Inflammatory subtype exhibited the highest proportion of TIDE-estimated responders (≈ 59.4%), followed by the IFN-γ Dominant subtype (≈ 35.8%), whereas the Wound Healing subtype showed the lowest response fraction (≈ 8.2%).

TIDE-derived indicators revealed divergent immunologic characteristics (**Figures 4B–D**, **S1**): the TIDE score was highest in Wound Healing and lowest in Inflammatory tumors, suggesting immune suppression in the former and immune activation in the latter; conversely, IFNG and Merck18 scores peaked in the IFN-γ Dominant subtype and were minimal in Wound Healing, consistent with strong interferon signaling and effector activation.

SubMap analysis further demonstrated that both IFN-γ Dominant and Inflammatory subtypes exhibited significant similarity to the PD-1-responsive reference group (nominal and Bonferroni-adjusted P < 0.05; **Figure 4E**), indicating distinct but convergent PD-1-sensitive immune programs within these subtypes.

Across multiple independent immunotherapy cohorts (GSE207422, GSE126044, GSE135222, and IMvigor210), the distribution of responders and survival outcomes consistently favored the Inflammatory and IFN-γ Dominant subtypes, while the Wound Healing subtype remained poorly responsive (**Figures 4F–K**). In GSE135222, clinical benefit (DCB vs. NDB) differed significantly among subtypes (Fisher P = 0.0482), with responders showing markedly prolonged PFS (P < 0.01). In IMvigor210, immune-subtype-mapped survival curves revealed pronounced separation (P = 0.004), and the Inflammatory (inflamed) subtype achieved the most favorable prognosis.

### 3.5 Establishment and validation of the DPIS (Differential Phenotype Immune Score) based on machine learning framework

Although both the Inflammatory and IFN-γ Dominant immune phenotypes are estimated to derive benefit from immunotherapy, they exhibit markedly divergent survival outcomes in the absence of treatment (**Figure 1D**). This observation motivated us to delineate the molecular determinants underlying this disparity. We first identified differentially expressed genes between these two subtypes and then performed univariate Cox regression to select survival-associated candidates. These candidates were subsequently entered into the Mime1 machine-learning framework for model development and internal validation (**Figure 5A**). Among all evaluated algorithms, the StepCox[forward] + elastic-net model (α = 0.1) yielded the most stable and reproducible performance across the TCGA-LUAD training cohort and five independent validation cohorts (GSE13213, GSE50081, GSE30219, GSE31210, and GSE42127), as suggesting by the highest mean C-index.

Based on this optimal model, we derived a composite risk score termed the Differential Phenotype Immune Score (DPIS). DPIS was defined as a Cox linear predictor calculated as DPIS = Σ(β_i_ × X_i_), where X_i_ denotes the log(x+1)-transformed expression of gene i and β_i_ represents the corresponding model coefficient learned from the TCGA training cohort. The complete coefficient table for all ten genes is provided in **Supplementary Data S3**, and the full implementation is available in our public code (GitHub repository: WakaWaka0419/LUAD_Immune_250825; Script 3). For risk stratification, the cutoff was prespecified in the TCGA training cohort (median DPIS) and then applied unchanged to each external cohort to avoid cohort-specific re-tuning.

Using this fixed scoring scheme, DPIS robustly stratified patients into high- and low-risk groups with significantly different overall survival across all six cohorts (**Figures 5B–G**; P = 0.003 in TCGA; P < 0.001 in GSE13213, GSE50081, GSE30219, and GSE31210; P = 0.0108 in GSE42127). Time-dependent ROC analyses further supported consistent discrimination at 1-, 3-, and 5-year time points (AUC range: 0.58–0.86), with several cohorts achieving short-term AUC values above 0.8 (**Figure 5H**). To complement discrimination-based metrics and assess potential clinical utility, we additionally performed decision curve analysis, which demonstrated a favorable net benefit of the DPIS-based model across clinically relevant threshold probabilities in the training and validation cohorts (**Supplementary Figure S3**).

Feature importance analysis identified ten recurrently selected genes (TPX2, UBE2C, CDC20, BIRC5, SCGB3A1, SFTPB, CACNA2D2, CYP4B1, MYBL2, and SUSD2), reflecting a combination of proliferation-associated programs and lung lineage–associated transcriptional features. Collectively, these results indicate that DPIS provides a stable, reproducible, and biologically interpretable prognostic framework across independent LUAD cohorts.

### 3.6 Single-cell validation and localization of DPIS origins and regulators

After projecting the bulk-derived DPIS (Differential Phenotype Immune Score) onto LUAD single-cell transcriptomes, we first visualized the distribution of major cell populations on the UMAP (**Figure 6A**, **S2**). DPIS appeared as a continuous gradient across cells (**Figure 6B**), allowing stratification into DPIS^+^ and DPIS^-^ subsets (**Figure 6C**). Composition analysis showed that tumor cells are markedly enriched in the DPIS^+^ subset (~44%), whereas their fraction is much lower in the DPIS^-^ subset (~15%) (**Figure 6D**); overall, DPIS^+^ cells constitute a minority but concentrate within the malignant compartment (**Figure 6E**). Focusing on malignant cells, DPIS levels were non-uniform across tumor states (**Figures 6F–H**): Proliferating Cancer cells exhibited the highest DPIS, followed by portions of the LAMC2/CXCL1 programs, whereas SOX2/CDKN2A programs showed comparatively lower scores (**Figures 6H, I**). Mapping “proliferation” and “DPIS^+^” separately revealed strong spatial overlap (**Figures 6J, K**); jointly defining DPIS^+^ Proliferating Cancer yielded a clearly clustered population in the tumor atlas (**Figure 6L**). Notably, DPIS program activity is not a proliferation-only surrogate: the DPIS-high malignant distribution does not colocalize with Ki-67 activity on the same UMAP embedding (**Supplementary Figures S4C, D**), indicating that DPIS captures a distinct malignant state beyond a Ki-67–driven cell-cycle program. Regulatory-network activity profiling (pySCENIC/SCENIC) identified regulons most specific to this DPIS^+^ proliferative state, with top candidates including ZNF443, ZNF429, ZNF92, NFE2, and E2F7 (**Figures 6M, N**), which display regionally elevated activity at single-cell resolution (**Figure 6N**). Collectively, DPIS primarily marks a high-proliferation, high-malignancy tumor-cell state at single-cell scale and is driven by distinct zinc-finger/E2F regulons.

### 3.7 Pan-cancer prognostic and functional landscape of TPX2

Building on our observation in **Figure 5I** that TPX2 ranked as the top shared survival-associated gene between the Inflammatory and IFN-γ Dominant immune subtypes—two groups that respond similarly to immunotherapy yet show striking differences in baseline survival—we performed a pan-cancer analysis to define its prognostic and biological relevance. Across 32 TCGA tumor types, TPX2 expression was broadly associated with adverse outcomes (**Figure 7A**); in LUAD and several other malignancies, higher TPX2 levels correlated with reduced overall survival (HR > 1, P < 0.05), supporting a conserved oncogenic role. A heatmap spanning four survival endpoints (OS, DSS, DFI, PFI) further confirmed a predominantly risky prognostic pattern of TPX2 across tumor contexts.

Functionally, correlation profiling in LUAD showed that TPX2 expression tightly aligned with cell-cycle and proliferative hallmarks—including E2F targets, G2M checkpoint, mitotic spindle, and MYC/mTORC1 signaling (**Figures 7B**, right)—while inversely correlating with metabolic programs such as bile acid and heme metabolism, consistent with a shift toward proliferation-oriented bioenergetics. TPX2 also exhibited negative associations with most immune-cell infiltration signatures (e.g., CD8^+^ T, NK, dendritic cells) but modest enrichment within fibroblast and endothelial lineages (**Figures 7B**, left), suggesting a cell-intrinsic, cell-cycle–driven tumor phenotype linked to an immune-cold milieu. Finally, among patients treated with immune checkpoint blockade, high TPX2 was generally associated with poorer overall and/or progression-free survival compared with TPX2-low cases, with concordant trends across cohorts (**Supplementary Data 2**).

### 3.8 TPX2 is aberrantly upregulated in lung cancer and functionally promotes tumor cell proliferation, migration and survival

**Figure 8:**
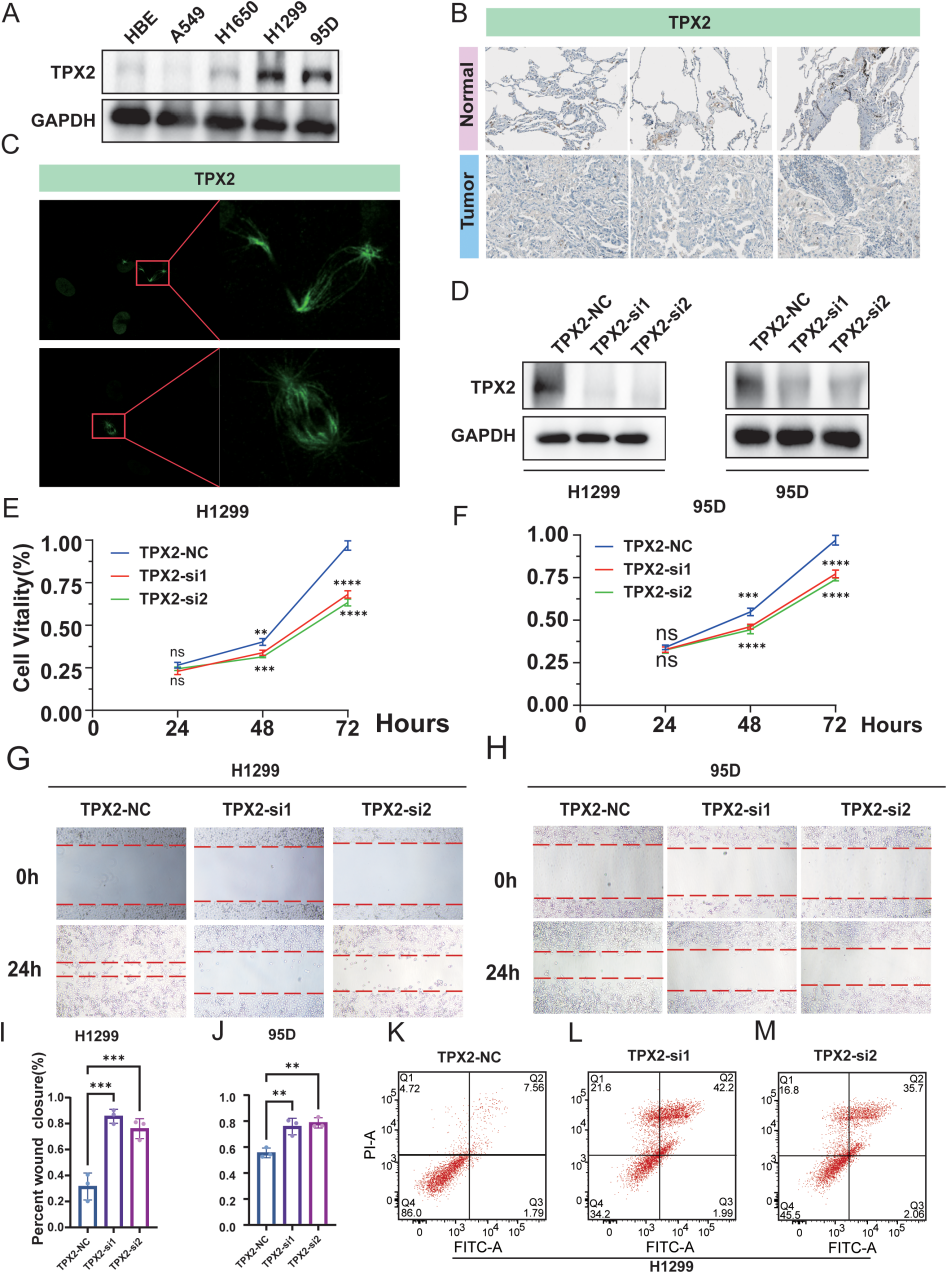
TPX2 is highly expressed in lung cancer and promotes proliferation and migration while suppressing apoptosis. **(A)** Western blot showing TPX2 expression in normal (HBE) and lung cancer cell lines. **(B)** Immunohistochemistry for TPX2 in normal lung and tumor tissues. **(C)** Immunofluorescence revealing TPX2 localization to mitotic spindle structures. **(D)** Knockdown efficiency of TPX2 via siRNAs in H1299 and 95D cells. **(E, F)** Cell viability assay after TPX2 knockdown at different time points. **(G, H)** Wound healing assay showing reduced migratory ability upon TPX2 silencing. **(I, J)** Quantification of wound closure. **(K–M)** Flow cytometry analysis of apoptosis in H1299 cells; TPX2 knockdown increases apoptotic cell populations. **P < 0.01; ***P < 0.001; ****P < 0.0001. “ns” stands for “not significant” (P ≥ 0.05).

To delineate the oncogenic relevance of TPX2 in lung cancer, we first profiled its expression across a panel of lung cancer cell lines. Immunoblotting revealed markedly elevated TPX2 levels in A549, H1650, H1299, and 95D cells, with minimal expression in non-malignant bronchial epithelial cells (HBE) (**Figure 8A**).

To extend these findings to patient-derived tissues, we interrogated immunohistochemistry data from the Human Protein Atlas (HPA). TPX2 protein levels were notably higher in lung tumor tissues compared to normal counterparts, with predominant nuclear and cytoplasmic localization in malignant cells (**Figure 8B**). Furthermore, immunofluorescence images from the HPA database demonstrated a mitosis-specific enrichment of TPX2 at spindle structures, consistent with its established role in spindle assembly and chromosomal alignment during cell division (**Figure 8C**).

Functional perturbation of TPX2 via siRNA-mediated knockdown in H1299 and 95D cells resulted in a robust reduction of TPX2 protein levels (**Figure 8D**). Time-course viability assays revealed a significant, progressive suppression of cell proliferation following TPX2 depletion (**Figures 8E, F**), indicating a proliferative dependency on TPX2 expression in these cell models.

We next assessed whether TPX2 modulates cellular motility. Scratch wound assays demonstrated markedly impaired migratory capacity upon TPX2 silencing in both H1299 and 95D cells (**Figures 8G–J**).

Finally, flow cytometric analysis using Annexin V/PI dual staining revealed a striking increase in apoptotic populations upon TPX2 knockdown (**Figures 8K–M**). The proportion of apoptotic cells rose from ~9% in control cells to over 40% in TPX2-silenced groups, implicating TPX2 in apoptotic resistance mechanisms.

Given the predicted link between TPX2 and immune evasion in our transcriptome-based analyses, we further performed immune-relevant functional assays and chemokine validation. In a T-cell co-culture system, TPX2 depletion significantly enhanced T-cell–mediated antitumor activity, as evidenced by increased cytotoxicity, elevated LDH release, and higher IFN-γ levels in the culture supernatant (**Supplementary Figure S5D–H**). In parallel, qPCR analysis revealed that TPX2 knockdown transcriptionally increased CXCR3-axis chemokines, with CXCL10 and CXCL11 showing consistent upregulation (while CXCL9 exhibited no significant change) across independent siRNAs (**Supplementary Figure S5A–C**). These findings support that TPX2 not only sustains malignant proliferation and survival but also contributes to an immune-unfavorable tumor state, at least in part by modulating T-cell–recruiting chemokine signals and tumor susceptibility to T-cell killing.

## 4 Discussion

Through integrative multi-omics, single-cell transcriptomic analysis, and machine learning modeling, this study delineates the molecular architecture of immune heterogeneity in lung adenocarcinoma (LUAD) and establishes a Differential Phenotype Immune Score (DPIS) to enable precise stratification of immune phenotypes. We identified three major immune subtypes—Wound Healing, IFN-γ Dominant, and Inflammatory—that differ markedly in immune infiltration, metabolic programming, and signaling activity. Moreover, we uncovered the cell cycle regulator TPX2 as a key determinant linking immune-cold phenotypes with poor prognosis. Collectively, these findings reveal a hierarchical organization of the LUAD immune ecosystem and bridge tumor-intrinsic signaling networks with functional states of the tumor immune microenvironment (TIME).

Immune heterogeneity has long been recognized as a central determinant of variable immunotherapy responses in LUAD. Here, the three identified immune subtypes form a continuous spectrum of immune activation and suppression. The Inflammatory subtype is characterized by abundant immune infiltration and robust cytokine signaling, consistent with an “immune-hot” microenvironment. In contrast, the IFN-γ Dominant subtype exhibits strong interferon signaling concomitant with upregulation of exhaustion markers, reflecting a paradoxical “hyperactivated yet suppressed” state. The Wound Healing subtype, enriched for TGF-β, WNT, and extracellular matrix (ECM) remodeling pathways, represents an immune-excluded phenotype. Similar patterns of immune stratification have been reported in recent multi-omics studies. For instance, Lian et al. identified two overarching immune classes—immune-cold and immune-inflamed LUAD—distinguished primarily by T-cell exhaustion and impaired antigen presentation. Together, these findings support the notion that LUAD immune ecosystems exist along a dynamically regulated continuum rather than as discrete static categories (37).

Our metabolic and signaling analyses further highlight the mechanistic basis of this immune heterogeneity. Divergent energy metabolism and transcriptional programs among subtypes suggest that metabolic plasticity is a key determinant of immune escape. The Wound Healing subtype demonstrates strong ECM remodeling and TGF-β activation, consistent with stromal barrier formation and angiogenic immune exclusion; the IFN-γ Dominant subtype exhibits heightened oxidative phosphorylation and ROS response, reflecting metabolic adaptation to immune stress; and the Inflammatory subtype activates NF-κB and lipid oxidation pathways to sustain immune-inflammatory balance. These results align with findings from Zhang et al (5)., who showed that sustained activation of mTORC1 and oxidative stress signaling drives immunosuppressive microenvironments and T-cell dysfunction. Our data extend this model to LUAD, suggesting that metabolic–immune crosstalk represents a conserved mechanism underpinning immune heterogeneity.

Mechanistically, TPX2 emerges as a pivotal molecular node in the establishment of immune-cold phenotypes. As a spindle assembly factor, TPX2 activates AURKA and drives the E2F/MYC transcriptional network, promoting accelerated cell-cycle progression and DNA replication (38). Multi-omics analyses revealed that TPX2-high tumors exhibit coordinated downregulation of MHC-I antigen presentation genes, suppression of interferon responses, and decreased chemokine signaling (39). These findings suggest that TPX2 orchestrates cell-cycle–dependent immune suppression through the AURKA–E2F–MYC axis. This hypothesis is supported by convergent evidence from recent studies. Wen et al. (40)demonstrated that hyperproliferative tumor cells show restricted antigen presentation and impaired immune recognition, while Nie et al. (41)reported that cell cycle–associated transcription factors can inhibit immune signaling cascades to facilitate immune evasion. Collectively, these studies reinforce the role of TPX2 as a functional bridge coupling proliferative signaling to immune suppression.

At the single-cell level, our findings provide direct evidence for this mechanism. High-DPIS cells predominantly overlap with highly proliferative tumor clusters and regions of active nuclear division, displaying synchronized expression of TPX2. These proliferative subpopulations are transcriptionally aligned with immune-cold phenotypes, characterized by diminished interferon activity and antigen processing. Such spatial and functional co-localization implies that part of LUAD’s immune heterogeneity arises from spatially constrained proliferative programs, wherein nuclear-proliferative tumor cells locally sculpt immunosuppressive niches. Similar spatial–functional coupling has been observed in other tumor contexts; for example, Ma et al. (42) showed that highly proliferative clones in NK/T-cell lymphoma induce immune exclusion through selective modulation of immune ligands. The recurrence of this pattern across tumor types provides compelling support for a unified model of proliferation-driven immune suppression.

From a translational perspective, the DPIS model offers a practical framework for stratifying LUAD patients with respect to immune responsiveness. High-DPIS tumors are characterized by TPX2 overexpression, limited immune infiltration, and poor clinical outcomes, whereas low-DPIS tumors display immune-activated transcriptional profiles consistent with higher ICI sensitivity. These results, together with growing evidence linking cell-cycle signaling to immune resistance (34, 43), suggest that pharmacologic inhibition of the TPX2–AURKA–E2F axis could reprogram proliferative immune-cold states and enhance checkpoint blockade efficacy.

In summary, this study integrates multi-omics and single-cell frameworks to uncover the layered mechanisms underlying LUAD immune heterogeneity. We propose a cell-cycle–driven immune suppression model, wherein TPX2 acts as a central regulatory hub linking proliferative signaling to immune evasion. The spatial overlap of high-DPIS and nuclear-proliferative tumor cells underscores the intimate connection between cell-cycle activity and local immune dysfunction. These findings provide new mechanistic insights into LUAD immunobiology and establish a rationale for combining cell-cycle–targeted agents with immunotherapy in precision oncology.

## 5 Conclusion

This study establishes an integrative framework for characterizing immune heterogeneity in lung adenocarcinoma (LUAD). By combining multi-omics profiling, single-cell transcriptomics, and machine learning, we identified three immune subtypes and developed the Differential Phenotype Immune Score (DPIS) to achieve precise immune stratification and prognostic prediction. We further reveal that TPX2, a key cell-cycle regulator, is strongly associated with immune-cold phenotypes and poor clinical outcomes, linking tumor proliferative activity to immune suppression. High-DPIS tumor cells exhibit enhanced proliferative signatures and reduced immune-related gene expression, suggesting that proliferative programs play a central role in establishing immunosuppressive states. Collectively, these findings provide mechanistic insight into the proliferative basis of LUAD immune heterogeneity and highlight TPX2 as a potential biomarker and therapeutic target for improving immunotherapy responsiveness.

## Data Availability

The original contributions presented in the study are included in the article/**Supplementary Material**. Further inquiries can be directed to the corresponding authors.

## Supplementary material

Supplementary Figure 1 Immune-related functional characteristics among LUAD immune subtypes. Boxplots illustrating immune-related functional scores across three immune subtypes of lung adenocarcinoma (LUAD): Wound Healing, IFN-γ Dominant, and Inflammatory. Shown features include MSI signature score, CD274 (PD-L1) score, CD8^+^ T-cell score, T-cell dysfunction score, immune exclusion score, MDSC score, CAF score, and TAM M2 macrophage score. Statistical significance is indicated as P < 0.05 (), < 0.01 (), < 0.001 (), and NS (not significant).

Supplementary Figure 2 Single-cell expression profiles of TPX2 and related marker genes in LUAD. Uniform Manifold Approximation and Projection (UMAP) plots showing single-cell transcriptomic expression patterns of TPX2 and selected marker genes in lung adenocarcinoma (LUAD). Genes include proliferation-associated markers (UBE2C, CDC20, BIRC5, MYBL2) and differentiation- or lineage-related markers (SFTPB, CYP4B1, SCGB3A1, CACNA2D2, SUSD2).
