# Supplementary figures and images for: Deciphering immune heterogeneity in lung adenocarcinoma via machine learning-based Differential Phenotype Immune Score: TPX2 as a key biomarker for immunotherapy resistance
Source: Front Immunol. 2026 Feb 27;17:1797282. doi: 10.3389/fimmu.2026.1797282 (PMC12982036; doi:10.3389/fimmu.2026.1797282)

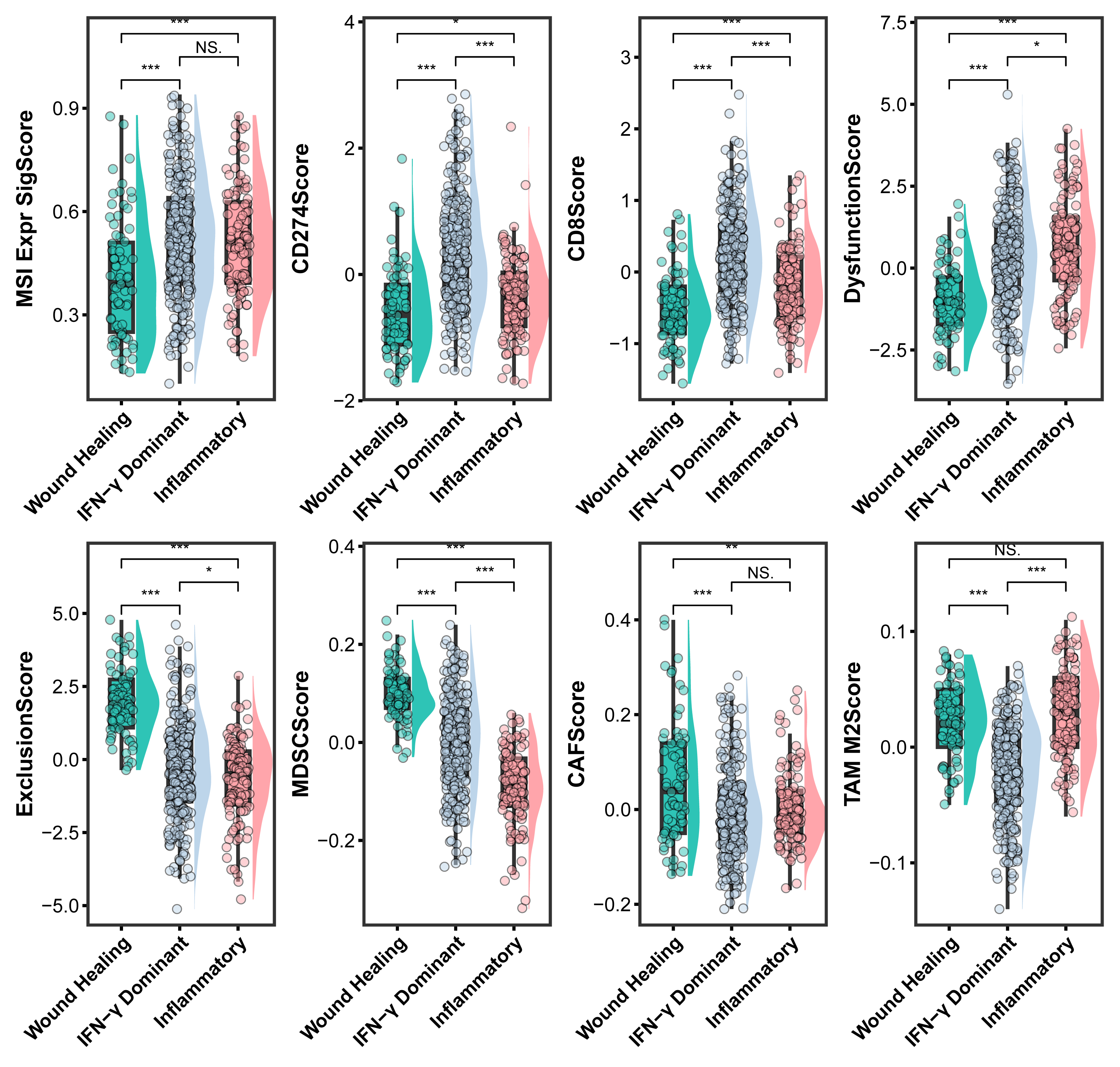

Supplement: Supplementary Figure 1 — Immune-related functional characteristics among LUAD immune subtypes. Boxplots illustrating immune-related functional scores across three immune subtypes of lung adenocarcinoma (LUAD): Wound Healing, IFN-γ Dominant, and Inflammatory. Shown features include MSI signature score, CD274 (PD-L1) score, CD8+ T-cell score, T-cell dysfunction score, immune exclusion score, MDSC score, CAF score, and TAM M2 macrophage score. Statistical significance is indicated as P < 0.05 (), < 0.01 (), < 0.001 (), and NS (not significant). [file Image1.tif]

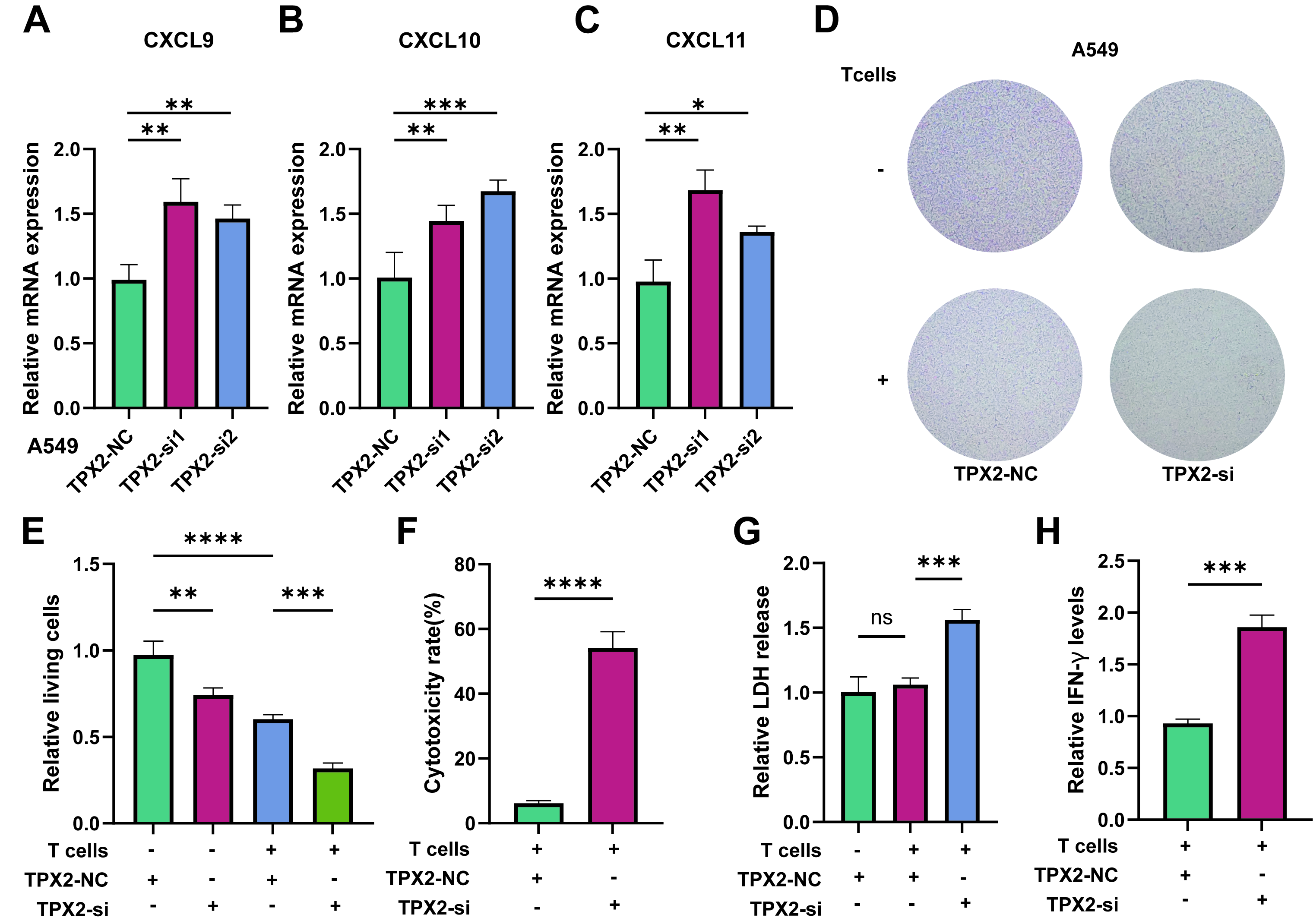

Supplement: Supplementary Figure 2 — Single-cell expression profiles of TPX2 and related marker genes in LUAD. Uniform Manifold Approximation and Projection (UMAP) plots showing single-cell transcriptomic expression patterns of TPX2 and selected marker genes in lung adenocarcinoma (LUAD). Genes include proliferation-associated markers (UBE2C, CDC20, BIRC5, MYBL2) and differentiation- or lineage-related markers (SFTPB, CYP4B1, SCGB3A1, CACNA2D2, SUSD2). [file Image2.tif]

TPX2

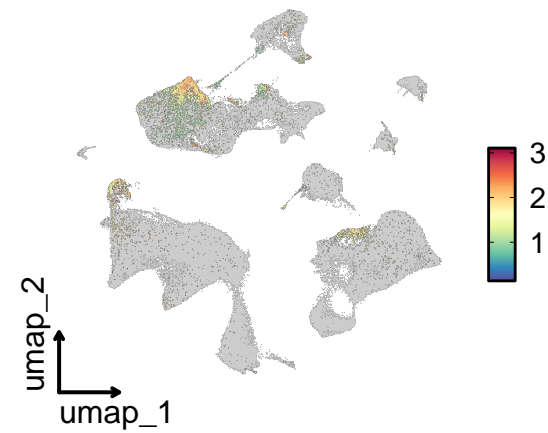

CYP4B1

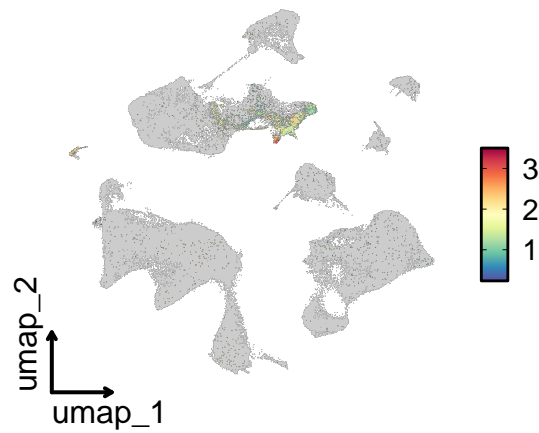

SCGB3A1

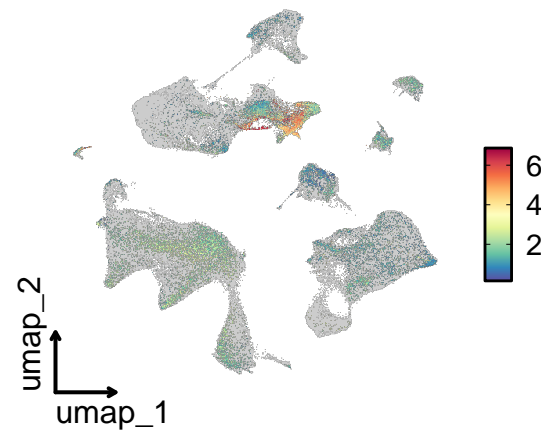

CACNA2D2

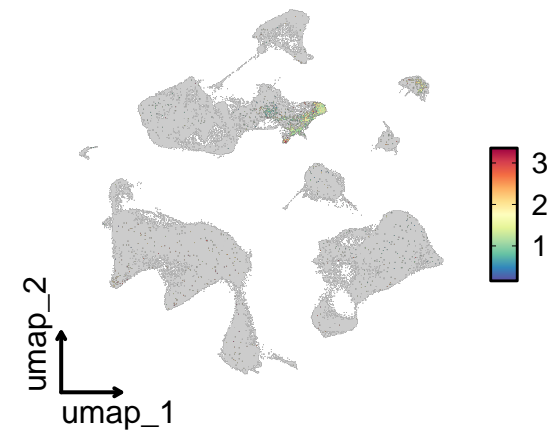

UBE2C

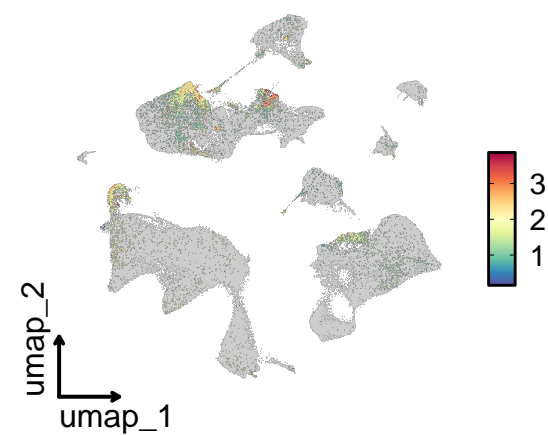

SFTPB

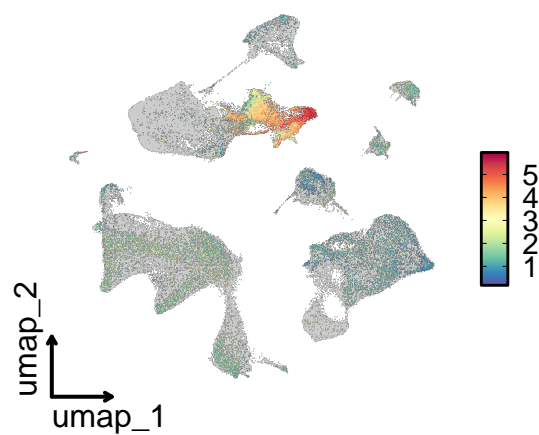

MYBL2

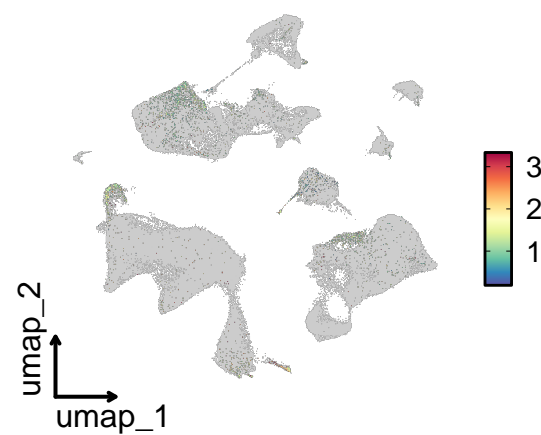

CDC20

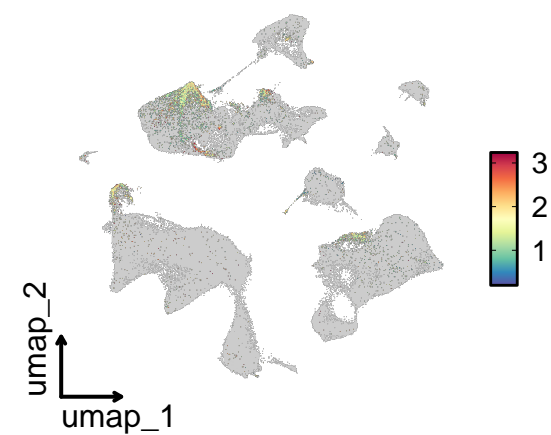

BIRC5

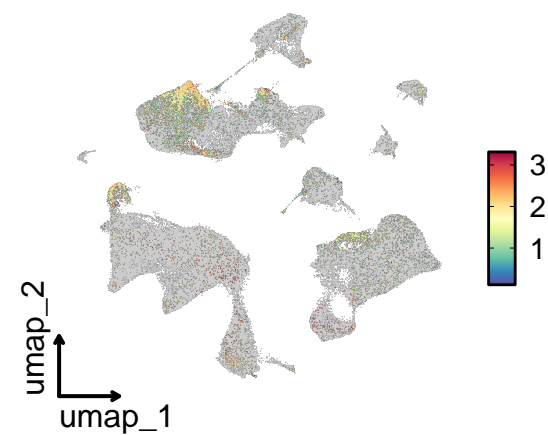

SUSD2

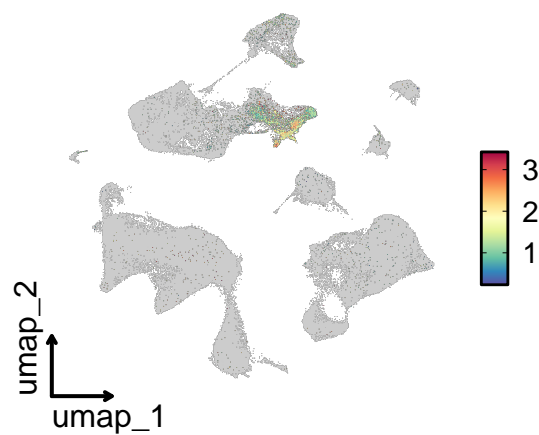

Supplement: Supplementary file 8 [file DataSheet5.pdf]

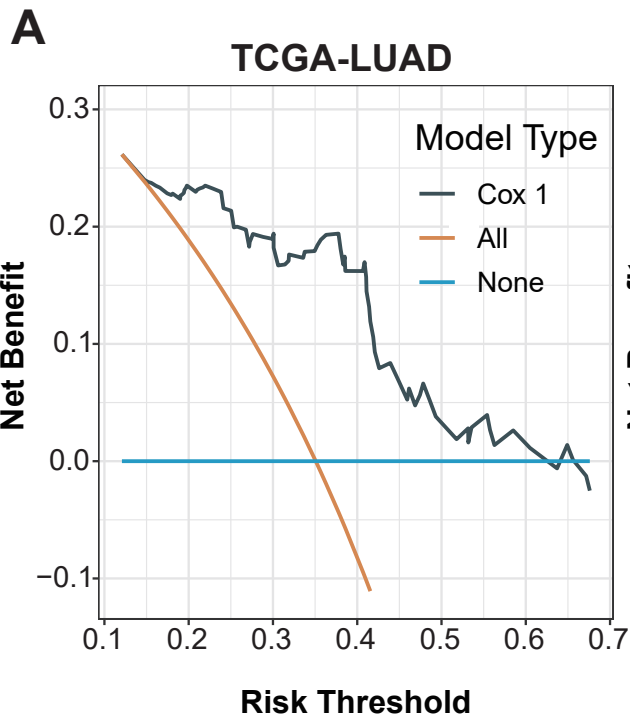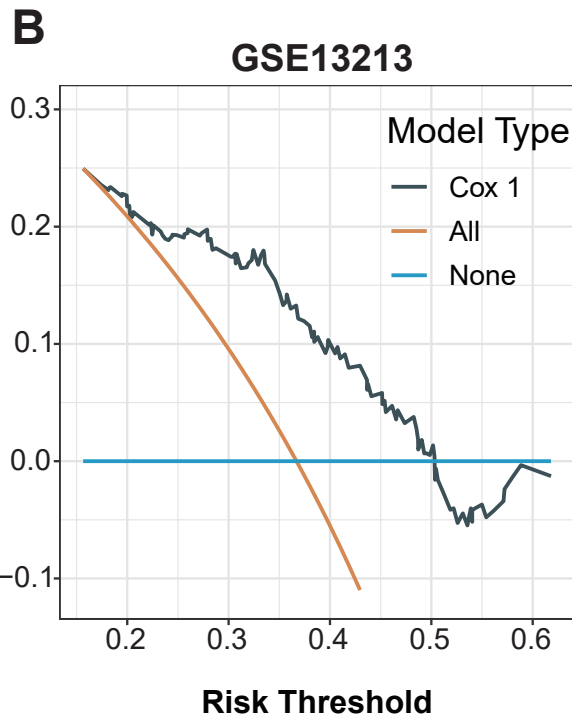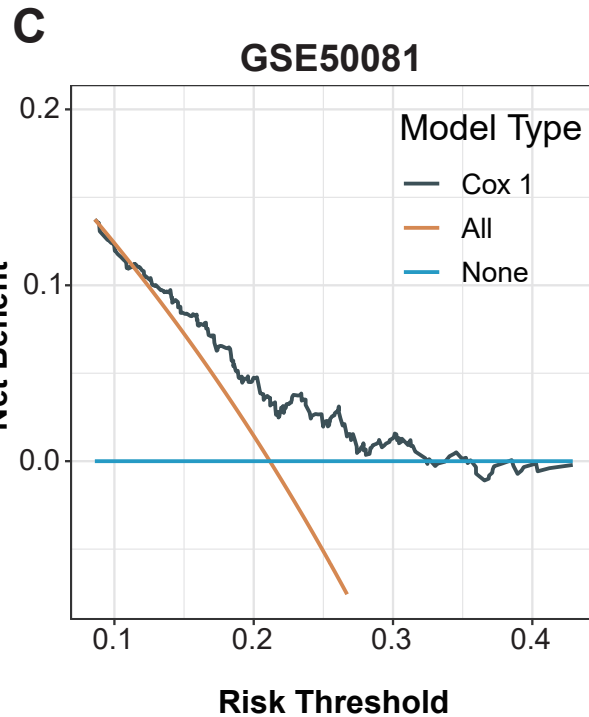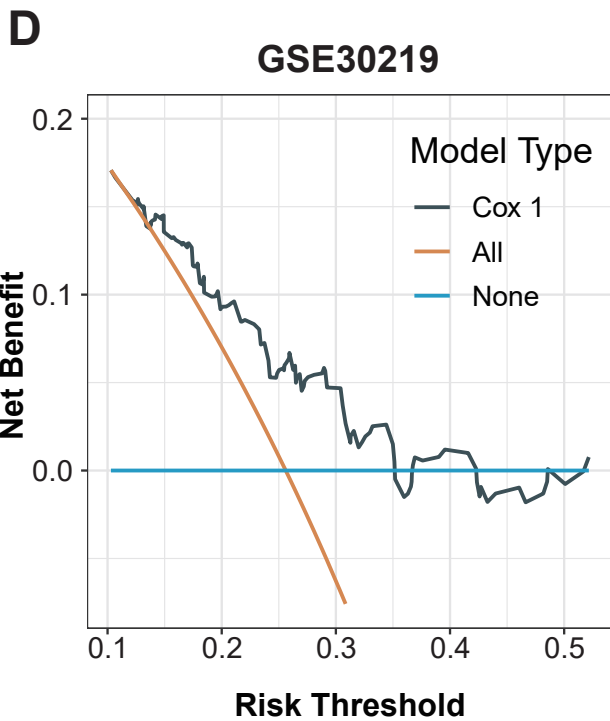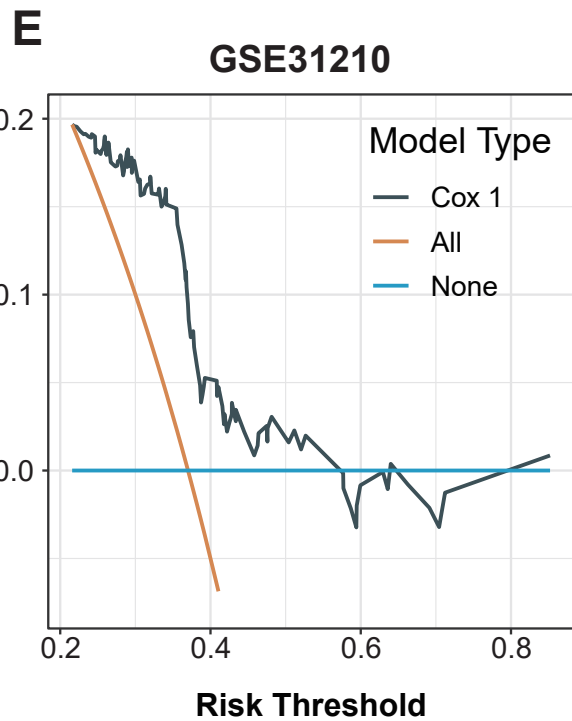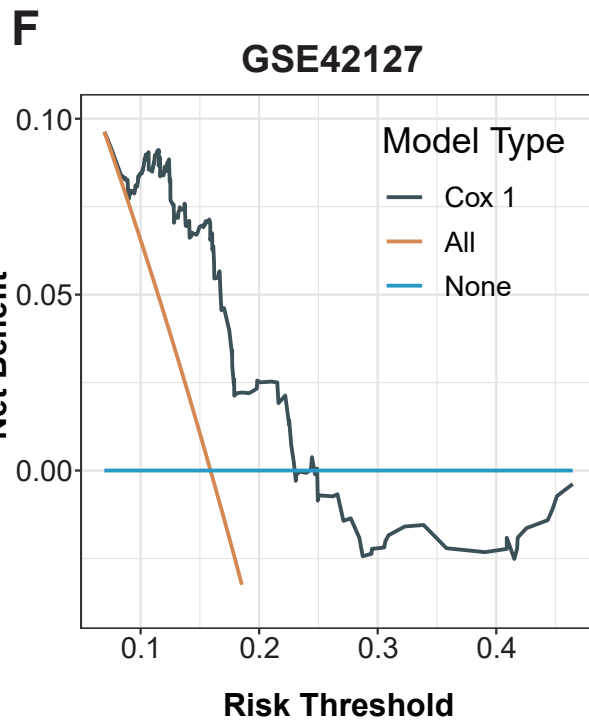

Supplement: Supplementary file 9 [file DataSheet6.pdf]

**A****Stage I and Stage II**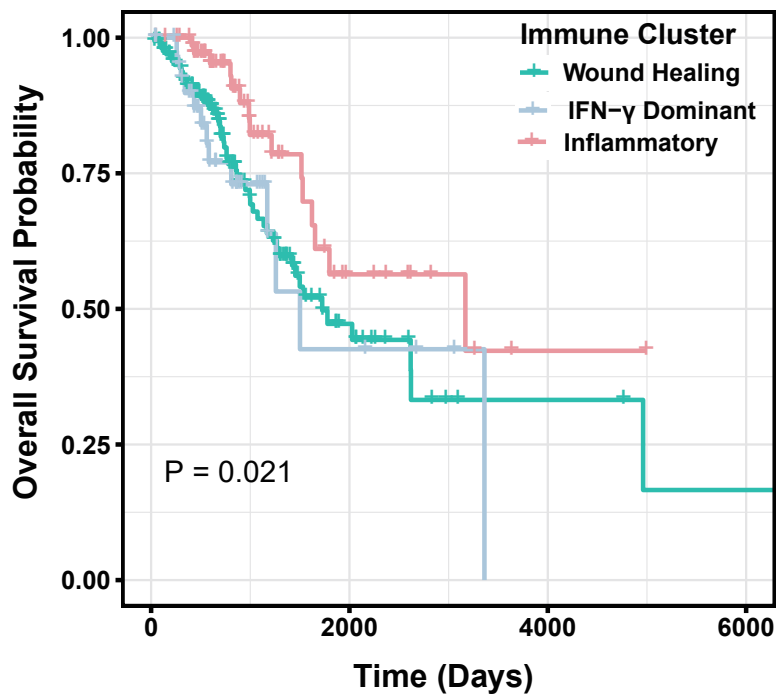**B****Stage III and Stage IV**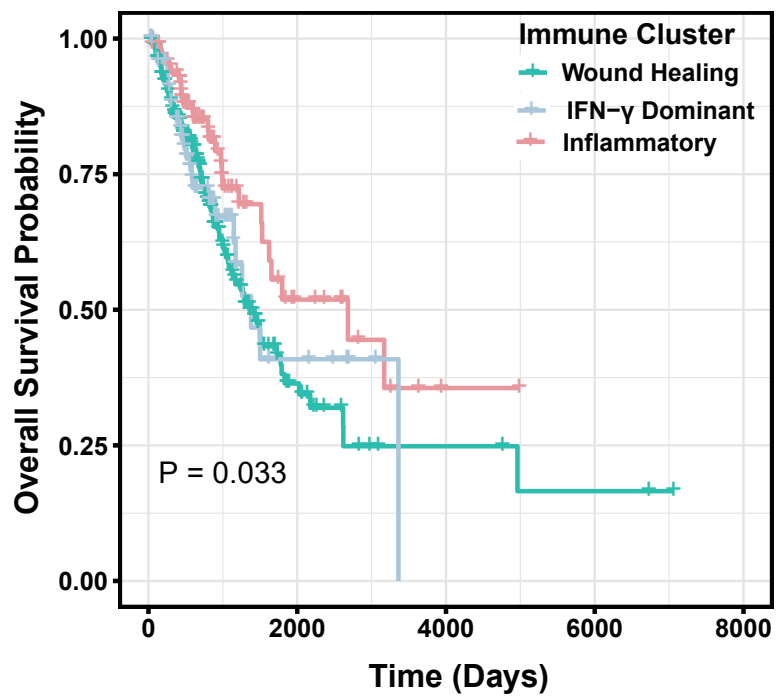**C****KI-67**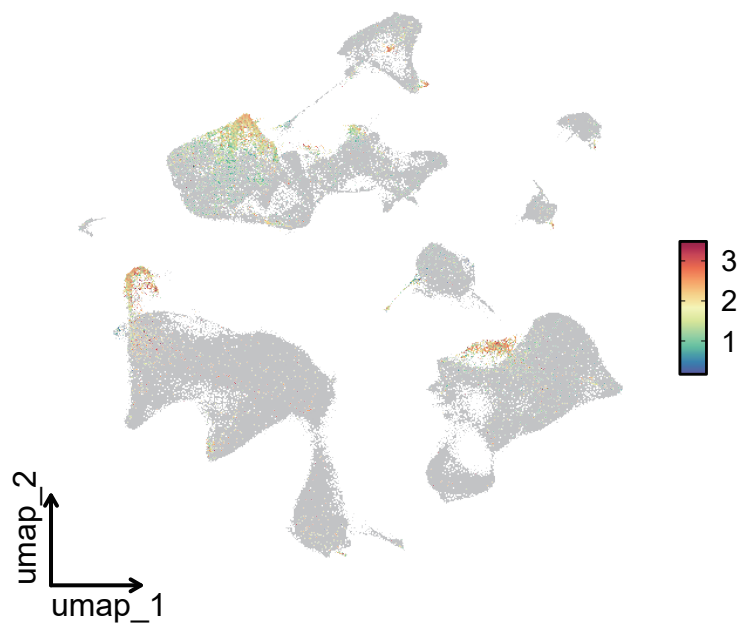**D****KI-67**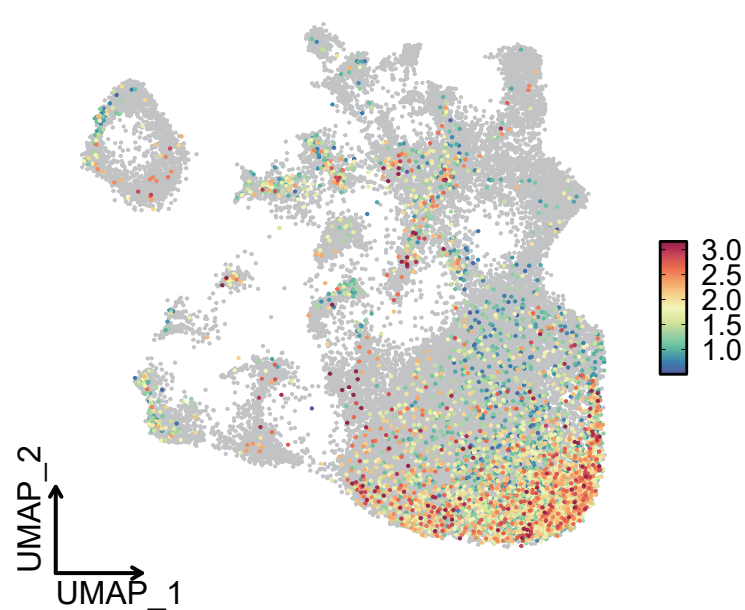

Supplement: Supplementary file 10 [file DataSheet7.pdf]
