# Supplementary material for: Deciphering immune heterogeneity in lung adenocarcinoma via machine learning-based Differential Phenotype Immune Score: TPX2 as a key biomarker for immunotherapy resistance
Source: Front Immunol. 2026 Feb 27;17:1797282. doi: 10.3389/fimmu.2026.1797282 (PMC12982036; doi:10.3389/fimmu.2026.1797282)
Supplement: Supplementary file 3 [file Table1.docx]

| **Oligonucleotides** | **Nucleotide sequence (5'-3')** |
| --- | --- |
| **siRNA** |  |
| Scramble control | GCUUCGCGCCGUAGUCUUA |
| Si-TPX2-1 | CCTGGAAGTTGAGGCAGCCATATCA |
| Si-TPX2-2 | TGGCTGGAATAGGGCAACCTGTGAA |
|  |  |

**Supplementary Table 1. Oligonucleotides used in research**
